# Integrated multi-optosis model for pan-cancer candidate biomarker and therapy target discovery

**DOI:** 10.3389/fbinf.2025.1630518

**Published:** 2025-09-19

**Authors:** Emanuell Rodrigues de Souza, Higor Almeida Cordeiro Nogueira, Ronaldo da Silva Francisco Junior, Ana Beatriz Garcia, Enrique Medina-Acosta

**Affiliations:** 1 Laboratório de Biotecnologia, Centro de Biociências e Biotecnologia, Universidade Estadual do Norte Fluminense, Rio de Janeiro, Brazil; 2 Pathology Department, Stanford University, Stanford, CA, United States

**Keywords:** cancer, multi-omics, multi-optosis, regulated cell death (RCD), signature database

## Abstract

Regulated cell death (RCD) is fundamental to tissue homeostasis and cancer progression, influencing therapeutic responses across tumor types. Although individual RCD forms have been extensively studied, a comprehensive framework integrating multiple RCD processes has been lacking, limiting systematic biomarker discovery. To address this gap, we developed a multi-optosis model that incorporates 25 distinct RCD forms and integrates multi-omic and phenotypic data across 33 cancer types. This model enables the identification of candidate biomarkers with translational relevance through genome-wide significant associations. We analyzed 9,385 tumor samples from The Cancer Genome Atlas (TCGA) and 7,429 non-tumor samples from the Genotype-Tissue Expression (GTEx) database, accessed *via* UCSCXena. Our analysis involved 5,913 RCD-associated genes, spanning 62,090 transcript isoforms, 882 mature miRNAs, and 239 cancer-associated proteins. Seven omic features—protein expression, mutation, copy number variation, miRNA expression, transcript isoform expression, mRNA expression, and CpG methylation—were correlated with seven clinical phenotypic features: tumor mutation burden, microsatellite instability, tumor stemness metrics, hazard ratio contexture, prognostic survival metrics, tumor microenvironment contexture, and tumor immune infiltration contexture. We performed over 27 million pairwise correlations, resulting in 44,641 multi-omic RCD signatures. These signatures capture both unique and overlapping associations between omic and phenotypic features. Apoptosis-related genes were recurrent across most signatures, reaffirming apoptosis as a central node in cancer-related RCD. Notably, isoform-specific signatures were prevalent, indicating critical roles for alternative splicing and promoter usage in cancer biology. For example, *MAPK10* isoforms showed distinct phenotypic correlations, while *COL1A1* and *UMOD* displayed gene-level coordination in regulating tumor stemness. Notably, 879 multi-omic signatures include chimeric antigen targets currently under clinical evaluation, underscoring the translational relevance of our findings for precision oncology and immunotherapy. This integrative resource is publicly available *via CancerRCDShiny* (https://cancerrcdshiny.shinyapps.io/cancerrcdshiny/), supporting future efforts in biomarker discovery and therapeutic target development across diverse cancer types.

## 1 Introduction

Regulated cell death (RCD) represents a highly controlled cellular process crucial for development, tissue homeostasis, and cellular stress responses (Newton et al., 2024). This process removes damaged, unnecessary, or potentially harmful cells, supporting organismal function and survival. RCD is essential in cancer research, playing dual roles in tumor suppression, progression and treatment resistance (Gong et al., 2023; Koren and Fuchs, 2021).

**FIGURE 1:**
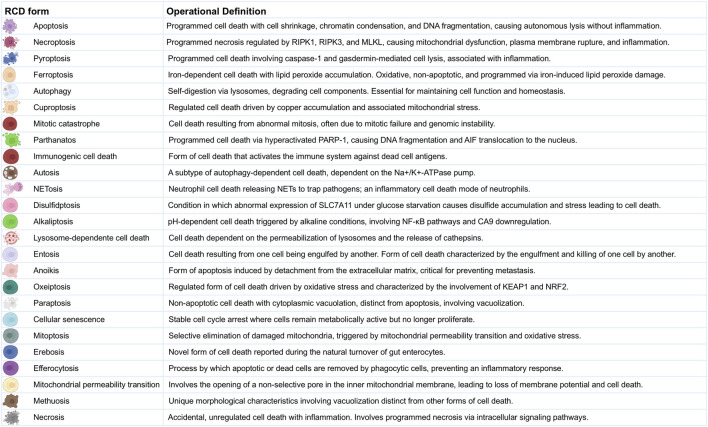
Operational Definitions of Regulated Cell Death Forms. This figure provides detailed operational definitions for the 25 RCD forms in the multi-optosis model. Each cell death process is characterized by specific biochemical and morphological features based on the Nomenclature of Cell Death 2018 (Galluzzi et al., 2018), with additional definitions derived from original research and reviews in 6,603 PDFs (corpus A).

RCD involves a complex network of signals and mechanisms from various cell death processes rather than functioning through a single, isolated pathway (Galluzzi et al., 2018; Ravel et al., 2020). The cell death processes are categorized into types, referred to as RCD forms, each playing distinct yet sometimes overlapping roles (Peng et al., 2022). The RCD forms include apoptosis (Elmore, 2007), necroptosis (Galluzzi et al., 2017), pyroptosis (Jorgensen et al., 2017), ferroptosis (Stockwell et al., 2017), autophagy (Debnath et al., 2023), cuproptosis (Feng et al., 2024), mitotic catastrophe (Castedo et al., 2004), parthanatos (Fatokun et al., 2014), immunogenic cell death (Choi et al., 2023), autosis (Bai et al., 2023), NETosis (Brinkmann et al., 2004), disulfidptosis (Zheng et al., 2023), alkaliptosis (Chen F. et al., 2023), lysosome-dependent cell death (Aits and Jaattela, 2013), entosis (Overholtzer et al., 2007), anoikis (Frisch and Francis, 1994), oxeiptosis (Holze et al., 2018), paraptosis (Sperandio et al., 2000), cellular senescence (Campisi, 2013), mitoptosis (Lyamzaev et al., 2020), erebosis (Ciesielski et al., 2022), efferocytosis (Qiu et al., 2023), mitochondrial permeability transition (Suh et al., 2013), methuosis (Maltese and Overmeyer, 2014), and necrosis (Kim et al., 2019). A summary of the operational definitions for the RCD forms is provided in Figure 1 and Supplementary Dataset S1A.

Most studies on RCD in cancer are confined to a death form (Liang et al., 2020; Zhang Y. et al., 2022; Zhang Z. et al., 2022; Xu et al., 2023). Multi-optosis, a growing concept describing the crosstalk between different RCD pathways, highlights the complexity of RCD in cancer. This interconnectedness can be exploited for therapeutic strategies that simultaneously induce multiple forms of cell death. Integrating various forms of RCD into explorative strategies to discover biomarkers has ranged from 3-optosis to 15-optosis models in a restricted number of cancer types (Su et al., 2023; Sun X. et al., 2024; Zou et al., 2022; Wei Q. et al., 2023; Wang and Zhang, 2024).

PANoptosis, a 3-optosis model, describes a unique inflammatory RCD pathway, characterized by a coordinated and often simultaneous convergence of features from pyroptosis, apoptosis, and necroptosis (Sun X. et al., 2024). It is thought to play a role in various physiological processes and diseases, including cancer (Samir et al., 2020; Wang and Kanneganti, 2021; Shi et al., 2023; Zha et al., 2023; Zhu et al., 2023). Research on the prognostic value of PANoptosis-related gene signatures in cancer is ballooning. In 2024 alone, the 3-optosis model has been assessed in a variety of cancers, including lung adenocarcinoma (Han et al., 2024), breast cancer (Yu et al., 2024), pancreatic adenocarcinoma (Zhao et al., 2024), hepatocellular carcinoma (Zha et al., 2023), colon adenocarcinoma (Liu et al., 2024), gastric cancer (Liu et al., 2024), head and neck squamous cell carcinoma (Gao et al., 2024), glioma (Sun F. et al., 2024), acute myeloid leukemia (Tang et al., 2024), thyroid cancer (Xie et al., 2024), and cutaneous melanoma (Zhong et al., 2023). Some models are mixed by including aging-associated and extrusion death-associated genes (Su et al., 2023).

A 12-optosis model, encompassing apoptosis, necroptosis, pyroptosis, ferroptosis, cuproptosis, entosis, NETosis, parthanatos, lysosome-dependent cell death, autophagy-dependent cell death, alkaliptosis, and oxeiptosis, was evaluated post-surgery in patients with triple-negative breast cancer (Zou et al., 2022). A 13-optosis model, including disulfidptosis, was assessed for lung carcinoma (Wei Q. et al., 2023). Recently, a 15-optosis model was assessed in postoperative bladder cancer patients (Wang and Zhang, 2024). This model encompasses pyroptosis, ferroptosis, necroptosis, autophagy, immunologic cell death, entosis, cuproptosis, parthanatos, lysosome-dependent cell death, intrinsic and extrinsic apoptosis, necrosis, and anoikis, as well as apoptosis-like and necrosis-like morphologies. The study identified a 13 gene-based cell death signature (*SFRP1*, *CDO1*, *HGF*, *SETD7*, *IRAK3*, *STEAP4*, *CD22*, *C4A*, *VIM*, *TUBB6*, *MFN2*, *FOXO3*, and *YAP1*).

Notably, the 13 genes contribute uniquely to the signature, each with distinct biological functions and associations with immune, tumor microenvironment, and clinical features, rather than sharing correlations across all phenotypic or genomic aspects to provide an overall prognostic score related to cell death in bladder cancer.

The discovery of molecular markers associated with RCD forms can serve as prognostic or predictive biomarkers, guiding treatment decisions and monitoring therapeutic responses (Zhou Y. et al., 2024). Targeting specific RCD forms can improve the effectiveness of current therapies. For example, in patients with chronic lymphocytic leukemia and acute myeloid leukemia who have relapsed or refractory disease, BH3 mimetics such as Venetoclax (ABT-199), designed to mimic the activity of BH3-only proteins, can sensitize cancer cells to apoptosis by inhibiting anti-apoptotic BCL-2 family proteins (Souers et al., 2013; Roberts et al., 2016; DiNardo et al., 2019).

Despite the diverse RCD forms, cancer cells often evade these processes through various mechanisms, including those involving cancer stem cells, which are the foundation of the disease (Hanahan and Weinberg, 2011). This evasion leads to unchecked proliferation and tumor development (Castelli et al., 2021). Many standard cancer treatments, including chemotherapy and radiation, aim to induce RCD in cancer cells. However, resistance to these treatments frequently arises from defects in RCD pathways. Mutations in genes regulating apoptosis, such as *TP53* and *BCL2*, are prevalent in various cancers and contribute to resistance to cell death and increased malignancy (Aubrey et al., 2018; Su et al., 2022). Mutations in genes critical for the execution of apoptosis, such as *CASP3* and *CASP9*, have been associated with various cancers, resulting in reduced efficacy of chemotherapy and radiation treatments (Ghavami et al., 2009). Mutations can inactivate apoptotic pathways or alter the expression of regulatory proteins, such as BCL-2 family members, contributing to multidrug resistance in cancer cells (Neophytou et al., 2021).

Research on identifying potential markers and therapeutic targets based on RCD forms in cancer often faces shortcomings. Most studies are limited to a single form of RCD (Liang et al., 2020; Zhang Y. et al., 2022; Zhang Z. et al., 2022; Xu et al., 2023), a specific type of cancer (Zou et al., 2022; Yu et al., 2024; Wei Y. et al., 2023; Chen et al., 2022), or a single type of association (i.e., mRNA expression *versus* T cell infiltrates and overall survival) (Zhu et al., 2023; Han et al., 2024; Wang X. et al., 2022). Studies often overlook the biological significance of whether correlations are positive or negative, the perturbances in gene expression compared to non-tumor samples, or rank the importance of gene signatures based on non-adjusted p-values rather than on a genome-wide scale (Pan B. et al., 2022; Gadepalli et al., 2021; Ye et al., 2023). Many reported gene expression signatures exhibit low correlation scores and limited clinical utility, raising questions about their effectiveness and reliability (Liang et al., 2020; Pan S. et al., 2022; Wu et al., 2021).

Unlike studies that assume uniform behavior of RCD-related genes across cancers, our approach acknowledges that each cancer type has its unique molecular and biological context. Thus, a gene that induces cell death in one cancer might help another cancer evade treatment. An example is *TP53*, which is commonly known to induce apoptosis in many types of cancer. Still, it has been found to promote survival in some contexts, depending on the cellular environment and specific mutations present (Aubrey et al., 2018). We thus recognize the non-uniformity in the involvement and roles of RCD-related genes across different RCD forms and cancer types. This non-uniformity means that the activities and effects of these genes can vary widely between different RCD forms and cancer types. By analyzing each gene and cancer type individually, we can understand these differences and identify multi-omic signatures that accurately capture the specific ways RCD-related genes contribute to each cancer. We believe this approach will lead to more precise biomarkers and better-targeted therapies.

Building upon the concept of multi-optosis, which describes the intricate crosstalk between distinct RCD pathways, our model integrates 25 forms of RCD into a comprehensive framework (Figure 1; Supplementary Dataset S1A) to enhance the identification of candidate biomarkers and potential therapeutic targets with genome-wide significance across multiple cancer types. The model provides a holistic view of RCD by analyzing multi-omic and phenotypic features as interconnected entities to understand their combined impact on cancer rather than studying each form independently. The identified signatures integrate clinically meaningful associations between multi-omic and phenotypic variables across 33 cancer types from The Cancer Genome Atlas (TCGA) Pan-Cancer analysis project (Cancer Genome Atlas Research et al., 2013), accessible through the UCSCXena portal^1^ (Goldman et al., 2020) and UCSCXena Shiny portal^2^ (Wang S. et al., 2022; Li S. et al., 2024). To facilitate data exploration and analysis, we developed two user-friendly tools: the *CancerRCDShiny* web browser (https://cancerrcdshiny.shinyapps.io/cancerrcdshiny/) and the Cancer Regulated Cell Death Data Analyst (https://chatgpt.com/g/g-8etzMPrtt-cancer-programmed-cell-death-data-analyst). These tools enable efficient extraction, analysis, and visualization of RCD data in cancer and related signatures, supporting a more effective interpretation of relevant data and enhancing the utility and impact of our findings.

To our knowledge, this is the first study to systematically map and classify Pan-Cancer signatures linked to 25 RCD modalities across seven omic layers, integrated with tumor phenotypic traits and clinical endpoints. In addition to conceptual innovation, we provide an interactive Shiny web application that enables real-time exploration of >44,000 multi-omic RCD signatures stratified by cancer type, omic modality, phenotype association, and survival relevance.

## 2 Materials and methods

### 2.1 Multi-optosis model specificities

The multi-optosis model integrates 25 forms of RCD (Figure 1, Supplementary Dataset S1A). Operational definitions of twenty forms of RCD followed the recommendations of the Nomenclature Committee on Cell Death 2018 (Galluzzi et al., 2018); RCD operational definitions not provided in the review by Galluzzi et al., 2018 were based on original research and reviews included 6,603 manually curated, free-access full-text PDF documents (Corpus A, Supplementary Material S1). We extracted, processed, and analyzed data on various forms of RCD and their associations with cancer using the PDF Ai Drive Tool^3^, which utilizes advanced large language models (LLMs) and natural language processing (NLP) techniques to extract and contextually analyze data. PDF AI Drive uses six AI models to summarize and extract structured information from PDF documents. The models are Claude 3 Haiku, Claude 3.5 Sonnet, Claude 3 Opus, CommandR+, Gemini 1.5 Flash and GPT-4o OpenAI (latest). GPT-4o provided us with the most detailed outputs.

A multi-optosis inventory of 5,913 genes was compiled by querying each RCD form term in the NCBI Gene database using a Boolean approach (Brown et al., 2015) (Supplementary Dataset S1B). The information was then programmatically extracted in R using the NCBI “*Entrez*” package. This approach solely reflects terms related to RCD forms and does not imply direct functional or causative involvement.

### 2.2 Signature construction: mono-omic, multi-phenotypic framework

Each signature in our study is designed as a mono-omic, multi-phenotypic construct. That is, a given signature is composed exclusively of one or more feature elements, derived from a single omic layer—either protein expression, somatic mutation, copy number variation (CNV), miRNA expression, transcript isoform expression, mRNA expression, or CpG methylation. We do not combine features from different omic layers within the same signature.

This design is guided by both biological rationale and computational feasibility. From a biological standpoint, each omic layer captures mechanistically distinct processes. Protein expression reflects post-translational modification and proteostasis; somatic mutations represent irreversible genomic alterations; CNV capture structural genome variation; miRNAs regulate gene expression post-transcriptionally; transcript isoforms result from alternative splicing; mRNA reflects transcriptional output; and methylation encodes epigenetic regulation. Merging these heterogeneous molecular signals into a single signature would conflate mechanistic interpretations and hinder clinical or biological inference.

Technically, the underlying data vary considerably in availability, granularity, and completeness across tumor types. RNA-Seq data (including mRNA, transcript isoforms, and miRNA) are nearly complete across TCGA cohorts. In contrast, RPPA protein expression covers only ∼258 targets with variable tumor representation, and DNA methylation profiles are probe-limited and sample-restricted. Mutation and CNV annotations also differ in resolution. A multi-omic integration would require imputation or sample filtering, introducing sparsity and reducing analytic robustness. By maintaining mono-omic integrity, each signature remains self-contained and biologically interpretable, while enabling systematic per-layer analysis across 33 cancer types.

Importantly, although each signature is mono-omic in structure, its phenotypic annotations—e.g., tumor vs non-tumor expression contrast, hazard ratio contexture (HRC), survival metric contexture (SMC), tumor microenvironment contexture (TMC), and tumor-infiltrating lymphocyte contexture (TIC)—may, when required, be inferred from mRNA-level or transcript isoform expression of the same gene locus. This bi-layer annotation strategy was specifically implemented for non-transcriptomic layers—protein, mutation, CNV, and methylation—when those layers lacked native support for phenotypic inference. For example, in methylation-specific signatures, TIC was assessed by the mRNA expression level of the gene bearing the CpG modification.

This bi-layer annotation logic is consistent with the expression-centric architecture of the UCSC Xena data model and reflects a pragmatic design constraint: we did not develop programmatic functions to compute HRC, SMC, TMC, and TIC directly from non-expression-based data such as mutations, CNV, or methylation profiles. We did implement transcript-based correlates for RPPA protein data due to its continuous expression-like structure, but this was not feasible for the categorical or sparse mutation, CNV, or methylation datasets.

This design choice is grounded in both biological plausibility and practical implementation considerations. Estimating phenotypic classifiers (e.g., immune infiltration, hazard ratios) reliably requires continuous, high-resolution, and biologically responsive signals—criteria met by RNA-based and protein-based datasets but not by mutation (sparse), CNV (categorical), or methylation (probe-limited) data. Furthermore, no established Pan-Cancer methodologies exist for computing immune or risk classifiers directly from these non-transcriptomic layers. Attempting such estimation would risk generating low-confidence or overfitted associations. Our approach thus prioritizes analytical rigor by applying a validated transcript-based phenotypic framework, while preserving the mono-omic identity of each signature and enhancing its biological interpretability.

An omic feature is incorporated into a signature if it reaches genome-wide significance for correlation with one of three key tumor-intrinsic variables: tumor mutation burden (TMB), microsatellite instability (MSI), or tumor stemness metric (TSM). These variables were analyzed in high-throughput mode across the genome and adjusted for multiple comparisons using the Holm–Bonferroni method (adjusted p < 5 × 10^−8^). All other phenotypic associations—namely survival endpoints, HRC, SMC, TMC, and TIC—were evaluated individually on a per-feature basis using univariate Cox regression or Pearson correlation and considered significant at unadjusted p < 0.05.

In the case of multi-element signatures, each feature included must share the same correlation direction for the phenotypic feature contexture (PFC), identical tumor vs non-tumor polarity, and common classification codes for HRC, SMC, TMC, and TIC. Features with divergent phenotypic patterns were split into separate signatures, each contextualized by its tumor type and phenotypic profile.

### 2.3 Correlation analysis between multi-omic and phenotypic variables in 33 cancer types

We conducted a comprehensive computational analysis correlating multi-omic variables with phenotypic outcomes from the TCGA Pan-Cancer analysis project (Cancer Genome Atlas Research et al., 2013), using primary datasets sourced from the UCSC Xena portal (Goldman et al., 2020), including the TCGA Pan-Cancer Atlas (Cancer Genome Atlas Research et al., 2013). Secondary datasets were obtained from the UCSC XenaShiny portal (Wang S. et al., 2022; Li S. et al., 2024), including the GTEx dataset^4^ for non-tumor tissue comparisons (Consortium et al., 2013).

The multi-omic feature included RNA-Seq transcriptomics (mRNA expression, transcript isoform expression, and miRNA expression), CpG methylation (450K array), CNV (gistic2 thresholded), mutations (SNP and INDEL; MC3 public version), and reverse-phase protein expression array (TCGA RPPA microarray) (Akbani et al., 2014; Sanjai et al., 2024). The microarray comprises 258 protein and modification probes relative to 210 genes, of which 239 are term-based associated with RCD forms (Supplementary Dataset S1C). miRNA gene symbols were converted to precursor miRNA identifiers (IDs) using “*BioMart*”^5^ (Ren et al., 2024), and the precursor IDs were converted to mature miRNAs (Supplementary Dataset S1D) using the “*miRBaseConverter”* R package^6^ to analyze miRNA. Gene symbols were converted to transcript IDs (Supplementary Dataset S1E) using the “*BioMart R*” package.

The phenotypic features included the patient’s indexes for TMB, MSI, TSM, hazard ratio, prognostic survival metrics, TMC and TIC. The term ‘HRC’ refers to the classification of omic signatures based on their prognostic association with survival outcomes in population-level Cox regression models. Each signature is assigned a categorical hazard classification code representing either an increased risk (risky), a decreased risk (protective), or no significant association across four survival endpoints. The signature’s HRC, derived from population-level Cox models, was integrated into the rank-based nomenclature system. The analysis was performed in R, using functions and customized source code based on the UCSC XenaShiny package (Wang S. et al., 2022; Li S. et al., 2024). These tools enabled us to execute multiple iterative analyses between multi-omic and phenotype programmatically features across 33 cancer types (n = 9,385 samples, Supplementary Dataset S1F).

To identify statistically significant associations, Holm–Bonferroni correction for multiple testing was applied exclusively to correlation analyses involving TMB, MSI, and TSM, which were conducted on a genome-wide scale across all omic features. Genome-wide significance was defined as an adjusted p-value <5 × 10^−8^. Once these significant omic feature elements were identified, subsequent associations with other phenotypic variables—including survival endpoints, as well as HRC, SMC, TME, and TIC—were evaluated individually for each signature. Because these phenotype associations were not derived from genome-wide correlation matrices, they were assessed using unadjusted p-values, with significance defined at p < 0.05.

For the comparison of mRNA expression between tumor and non-tumor tissues, including primary-tissue-matched samples from the GTEx project (n = 7,429 samples, Supplementary Dataset S1F), we use the Wilcoxon test (Consortium et al., 2013). This nonparametric test was selected to handle potential deviations from normality in the expression data, ensuring robust comparative analysis.

For tumor *versus* non-tumor expression analyses, gene- and isoform-level RNA-Seq data were obtained from the UCSC Xena public repository (Goldman et al., 2020; Wang S. et al., 2022; Li S. et al., 2024), which hosts uniformly processed expression data from both TCGA tumor tissues and GTEx normal samples. These datasets were derived from the UCSC Toil RNA-Seq Recompute pipeline (Vivian et al., 2017), which implements a consistent processing workflow for TCGA and GTEx RNA-Seq data and includes batch correction, normalization, and expression quantification under identical conditions. As a result, technical confounding because of cross-cohort differences was minimized, allowing for valid comparisons between tumor and non-tumor profiles. The datasets were retrieved using the UCSCXenaShiny application (Wang S. et al., 2022; Li S. et al., 2024).

TCGA *versus* GTEx tissue RNA-Seq expression profiles were classified as unchanged, underexpressed, overexpressed, or with no data. Unchanged expression includes genes with a *padj*-value ≥0.05. Genes are classified as overexpressed or underexpressed if they have a *padj*-value <0.05. Overexpressed genes show higher median expression in tumor tissue, while underexpressed genes show lower median expression in tumor tissue, both compared to non-tumor tissue.

We performed hazard ratio analysis using the Cox proportional hazards regression model to assess the prognostic significance of the association between omic variables and patient survival outcomes, providing hazard ratios that refer to the relative risk of events occurring at any given point in time. Univariate Cox proportional hazards models were used to estimate the association between each omic feature or signature and survival outcomes. Standard clinical covariates (e.g., age, sex, tumor stage) were not included at this discovery phase, as the objective was to enable large-scale, systematic signature discovery across omic layers and cancer types. Expanding the model to include covariate-adjusted effects would require redefinition of the signature elements to retain correlation within each subgroup, and the construction of stratified indices across clinical layers within the Xena-derived framework. We acknowledge this as a valid direction for future validation studies.

Multi-omic features with consistent correlations, showing the same direction in tumor versus non-tumor expression, and Cox hazard ratio were used to create signatures. These signatures were then evaluated individually by summing the values of the constituent features (i.e., member 1 + member 2 + … + member n). The prognostic significance of the constructed signatures was evaluated using Cox proportional hazards analysis for four survival metrics: Disease-Specific Survival (DSS), Disease-Free Interval (DFI), Progression-Free Interval (PFI), and Overall Survival (OS). Kaplan-Meier survival curves were generated for each metric, and log-rank tests were applied to compare survival distributions across patient groups, determining the statistical significance of observed differences. Together, these survival analyses offer a comprehensive view of patient outcomes and provide valuable insights into the effectiveness of cancer treatments (Royle et al., 2023). The survival metrics are defined: DSS specifically measures survival without death attributed to the cancer being studied. It provides a more focused measure of treatment effectiveness on the targeted disease. DFI assesses the period after treatment during which the patient remains free from any signs or symptoms of cancer. It is helpful in evaluating the efficacy of therapies. PFI measures the duration in which the cancer does not progress or worsen. OS is a critical endpoint in cancer clinical trials, measuring the time from randomization or diagnosis to death from any cause. It is the most definitive endpoint, reflecting the ultimate impact of the treatment on patient survival (Korn and Crowley, 2013).

### 2.4 Classification of signatures according to the tumor microenvironment profile

We used CIBERSORT (Cell-type Identification By Estimating Relative Subsets Of RNA Transcripts) (Newman et al., 2015) and xCell (Aran et al., 2017) deconvoluted bulk gene expression data from UCSCXenaShiny (Wang S. et al., 2022; Li S. et al., 2024) to estimate correlations of the multi-omic gene-signature feature and the cellular composition of complex tissues based on 29 predefined immune cell signature subsets, including B cells (naïve, memory, plasma, class-switched memory), T cells (CD8^+^, CD4^+^ naïve, CD4^+^ memory resting, CD4^+^ memory activated, CD4^+^ Th1, CD4^+^ Th2, follicular helper, regulatory Tregs, gamma delta), NK cells (resting and activated), monocytes, macrophages (M0, M1, M2), myeloid dendritic cells (resting and activated), activated mast cells, eosinophils, neutrophils, cancer-associated fibroblasts, common lymphoid progenitor, endothelial cell, granulocyte-monocyte progenitor, and hematopoietic stem cell.

We categorized the signatures as anti-tumoral, pro-tumoral, or dual with respect to tumor progression. This classification was based on the Spearman correlation coefficients between mRNA, miRNA, isoform RNA-Seq or protein expression of the signature database and the RNA-Seq expression profiles of the 29 specific cell infiltrate types representative of the tumor microenvironment profile (Supplementary Dataset S1G). We used the categorizations “hot,” “cold,” and “variable” for the involvement of cell infiltrates, based on evidence from the literature (Supplementary Dataset S1G).

In this system, the signs and magnitudes of the correlation coefficients provide insights into different tumor microenvironment scenarios (See Supplementary Figure S1 for the categorization framework of tumor microenvironments and immune phenotypes across multiple scenarios). A positive correlation with a cell type shows a higher presence of that cell type in the tumor microenvironment for signatures that are overexpressed in a tumor type. Conversely, for underexpressed signatures, a positive correlation with a cell type shows a lower presence of that cell type. For overexpressed signatures exhibiting a negative correlation, the correlation sign also shows a lower presence of that cell type. Similarly, underexpressed signatures with a negative correlation show a higher presence of that cell type. For signatures whose expression profiles are unaltered between tumor and non-tumor tissues, a positive correlation indicates the presence of cell infiltrates, while a negative correlation indicates their absence.

We combined the correlation coefficients for all cell types to classify the signatures according to the tumor microenvironment, considering their signs. Signatures with the highest combined magnitude for anti-tumoral cell types were classified as anti-tumoral. Similarly, signatures with the highest combined correlations for pro-tumoral cell types were classified as pro-tumoral, and signatures with the highest combined correlations for dual microenvironment cell types were classified as dual. Detailed methodology is provided in Supplementary Material S1 (Methodology 1).

### 2.5 Classification of signatures according to the tumor immune phenotype

The tumor immune phenotype, classified as hot, cold, or variable based on immune cell infiltration, guides therapeutic interventions and identifies patients who are resistant to immunotherapies (Galon and Bruni, 2019; Wang L. et al., 2020). Hot tumors exhibit high levels of cytotoxic T cells (NK and CD8^+^) and M1 macrophage signatures, while cold tumors show low T cell infiltration, a predominance of M2 macrophages, and immunosuppressive cells. Variable tumors have intermediate characteristics (Supplementary Dataset S1H). Examples include melanoma and lung cancer as hot, and prostate and pancreatic cancers as cold. Immune checkpoint inhibitors are more effective in hot tumors (Galon and Bruni, 2019; Wang L. et al., 2020). Strategies to convert cold and variable tumors to hot ones, such as nanomedicines and combination therapies, are under development (Wang M. et al., 2020).

We employed a classification method analogous to immunohistochemistry as a proxy to quantify tumor lymphocyte infiltration using RNA-Seq indexes (Aran et al., 2017; Galon and Bruni, 2019; Wang M. et al., 2020), allowing for categorization into “hot”, “cold”, or “variable”. This enables the automated categorization of signatures as “hot”, “cold”, or “variable” in R, thereby enhancing the understanding of tumor immunological characteristics and potential responses to immunotherapies. “Hot” tumors correlate positively with cytotoxic T cells and M1 macrophages, while “cold” tumors show low correlations with these cells but high correlations with M2 macrophages and Tregs. “Variable” tumors exhibit intermediate correlations (Supplementary Dataset S1H).

For classification, we used Spearman correlation coefficients and p-value significance to analyze the relationship between RNA-Seq-based expression profiles of signatures and immune cell profiles (T CD8^+^, NK, M1/M2 macrophages, and Tregs) (Galon and Bruni, 2019; Wang L. et al., 2020). In ambiguous cases, we applied a differentiated weighting criterion, prioritizing CD8^+^ T cells and NK cells because of their importance in classifying “hot” tumors and predicting immunotherapy responses. Detailed methodology is provided in Supplementary Material S1 (Methodology 2).

### 2.6 Multi-optosis and multi-omic signature nomenclature

**FIGURE 2:**
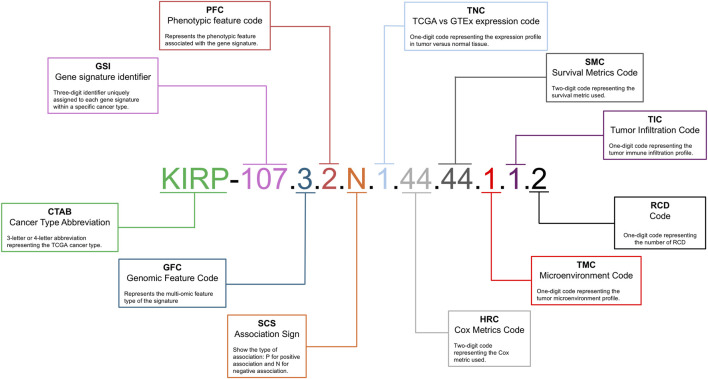
Multi-omic Signature Nomenclature and Coding System. This figure details the nomenclature and coding system used for multi-omic signatures in the multi-optosis model. Each signature is uniquely identified by a series of codes that represent different attributes: the cancer type abbreviation is a three- or four-letter abbreviation denoting the TCGA cancer type (i.e., KIRP for kidney renal papillary cell carcinoma); the phenotypic feature code is a one-digit code showing the specific phenotypic feature associated with the signature; the genomic feature code is a one-digit code representing the multi-omic feature; the signature identifier is a unique three-digit number assigned to each signature within a specific cancer type; the correlation sign shows the type of association, with ‘P' for positive and ‘N' for negative; the TCGA *versus* GTEx expression code is a one-digit code showing the gene expression profile in tumor tissue compared to non-tumor tissue; the Cox metrics code is a two-digit code representing the Cox proportional hazards metric used in the analysis; the survival metrics code is a two-digit code showing the specific survival metric applied; and the tumor infiltration code is a one-digit code representing the tumor immune infiltration profile. An example of a multi-omic signature identifier, such as KIRP-107.3.2.N.1.44.44.1.1.2, illustrates how these codes combine to form a comprehensive identifier for each signature. This standardized coding system enables precise classification and analysis of signatures in cancer research.

The signature nomenclature system provides a structured alphanumeric identifier that categorizes signatures derived from multi-omic Pan-Cancer analysis. This system links the multi-omic features of target genes with phenotypic characteristics across 33 cancer types, ensuring high precision and clarity in data organization and retrieval. The signature identifier follows an eleven-component structure: **CTAB-GSI. GFC.PFC.SCS.TNC.HRC.SMC.TMC.TIC.RCD** (i.e., KIRP-107.3.2.N.1.44.44.1.1.2) (Figure 2).

Each component is defined as:

**CTAB** refers to a 3- or 4-letter abbreviation representing the cancer type from the TCGA database (i.e., KIRP for kidney renal papillary cell carcinoma; see Supplementary Dataset S1I for cancer type abbreviations).

**GSI** is a 1- to 4-digit identifier (i.e., 107) unique to each signature within a cancer type.

**GFC** represents the genomic feature contexture of the signature: 1 for Protein expression, 2 for Mutations, 3 for CNV, 4 for miRNA expression, 5 for Transcript expression, 6 for mRNA expression, and seven for CpG Methylation.

**PFC** denotes the phenotypic feature contexture linked to the signature: 1 for TMB, 2 for MSI, and 3 for TSM.

**SCS** shows the Spearman Correlation Sign: P for positive and N for negative correlations.

**TNC** represents tumor *versus* non-tumor tissue expression contexture: 0 for no data, 1 for unchanged expression, 2 for underexpressed, and 3 for overexpressed.

**HRC** stands for Hazard Ratio contexture, represented as the alphanumeric array 1N2N3N4N. This shorthand notation encodes the significance levels of multiple survival metrics. The digits 1 to 4 correspond to the survival metrics: DSS, DFI, PFI, and OS, respectively. The letter N denotes the hazard effect, classified as A (no effect), B (risky), or C (protective).

**SMC** is the Kaplan-Meier survival distribution contexture across patient groups. It also follows the array 1N2N3N4N, where the digits 1 to 4 correspond to survival metrics: DSS, DFI, PFI, and OS, respectively. However, the categorization of the letters A, B, C, and D across multi-omic features reflects distinct classifications based on specific criteria. The letter A is used universally for all omic layers (Protein, Mutation, CNV, miRNA, Transcript, mRNA, and Methylation) when the category is “NS” (Not Significant).

The letter B varies according to the omic layer. For Protein, miRNA, Transcript, mRNA, and Methylation, it corresponds to the category “High”. For the Mutation feature, B represents “MT” (Mutant), while for the CNV feature, B refers to “Deleted.” Similarly, the letter C also differs by omic layer. For Protein, miRNA, Transcript, mRNA, and Methylation, C corresponds to the category “Low.” For the Mutation feature, it represents “WT” (Wild Type), and for CNV, it reflects the “Duplicated” status.

The letter D is used explicitly for the CNV feature and represents the category “Deleted/Duplicated,” which encompasses both deletion and duplication events.

There are 128 combinations of the 1N2N3N4N array for hazard values and survival metrics. Each array combination is reassigned to a specific numerical identifier ranging from 0 to 127 (Supplementary Dataset S1J). For instance, 1A2A3A4A (no effect for DSS, DFI, PFI, and OS) is reclassified to the identifier 0. In contrast, 1A2A3A4B (no effect for DSS, DFI, and PFI, yet “risky” for OS) is reclassified accordingly under the identifier 1.

**TMC** refers to the Tumor Microenvironment contexture: 1 for anti-tumoral, 2 for dual, 3 for pro-tumoral, and 4 for no significant data.

**TIC** is the tumor-infiltrating lymphocyte contexture, which defines immune cell infiltration: 1 for “hot”, 2 for “variable”, 3 for “cold”, and 4 for no significant data.

**RCD** is a 1- to 2-digit code representing the number of RCD forms linked to the signature.

### 2.7 Signature rank method

We developed the *Cancer Multi-optosis Multi-omic Signature Rank Calculator in R* to evaluate how effectively a signature provides valuable, actionable insights to improve patient care or inform clinical decisions—its clinical meaningfulness potential—within our Pan-Cancer multi-optosis and multi-omic model. This system ranks candidate biomarker signatures by integrating multi-omic and phenotypic identifiers. Each component within a signature is assigned an integer rank based on its importance in predicting patient outcomes, such as survival prognosis (Liu et al., 2018) and immunotherapy potential. The immunotherapy potential is assessed using TME and TIC identifiers, applying the concepts of immune “hotness” and “coldness,” which reflect the level of immune infiltration in tumors (Galon and Bruni, 2019; Wang L. et al., 2020). A rank is assigned to each signature component through a mapping function in R, which attributes integer values to multi-omic and phenotypic identifiers. The final rank for each signature is obtained by summing the ranks of its individual components. Detailed criteria for assigning ranking values are provided in Supplementary Material S1 (Methodology 3).

### 2.8 Drug-gene interaction analysis

To identify potential therapeutic targets, we conducted a comprehensive cross-referencing analysis of gene components from the top-ranked multi-modular and clinically meaningful signatures. The gene members of these signatures were queried against the Drug–Gene Interaction Database (DGIdb 5)^7^ (Cannon et al., 2024), which integrates drug-gene interaction and druggability data from multiple sources, facilitating the exploration of potential pharmacological interventions.

To construct the drug-gene interaction network, we retrieved curated interaction data from DGIdb 5.0, excluding undefined or unknown interaction types to ensure the identification of meaningful associations. The dataset was processed in R using the “*tidyverse”* suite, which included data cleaning, removal of redundant entries, and standardization of gene and drug names. A bipartite network was generated, where genes (from top-ranked multi-omic RCD signatures) formed one node type, and drugs (categorized by interaction type) formed the other node type. The edges in the network represent drug-gene interaction relationships, as defined by DGIdb. Network visualization was performed using *“igraph*” and *“ggraph”* for static rendering, with the Fruchterman-Reingold force-directed layout applied to optimize node distribution and improve clarity.

### 2.9 Validation using the independent PRECOG cancer database

To validate the prognostic value of the selected mRNA-specific signatures associated with risk, protection, and poor prognosis, we used the PRECOG (PREdiction of Clinical Outcomes from Genomic Profiles) database^8^ (Gentles et al., 2015). PRECOG is a curated resource that provides a standardized meta-analysis framework to generate prognostic meta-Z scores, which quantify the strength and direction of the association between gene expression and OS across multiple cancer types. The database integrates transcriptomic data from publicly available datasets, encompassing 28 cancer types independent of TCGA but equivalent to 24 TCGA cancer types (Supplementary Dataset S1I). Meta-Z scores were extracted from the PRECOG repository for each gene within the 126 signatures selected for their association with risk or protection in all survival metrics and with anti-tumoral, pro-tumoral, or dual microenvironment cell profiles, as well as hot, cold, or variable immune infiltrates (Supplementary Dataset S1K). To validate significantly poorer or better prognosis associations, the validation process relied on stringent statistical thresholds (|Meta-Z| > 3.09 or < −3.09, p < 0.001). This validation set of signatures represents only 11 TCGA cancer types (ACC, BLCA, BRCA, CESC, HNSC, KIRP, LGG, LUAD, LUSC, PRAD, STAD). For single-gene signatures, the corresponding meta-Z score was retrieved for each cancer type. For multi-gene signatures, each gene was queried individually, and the median meta-Z score across all genes was computed to derive the final signature-level score. To identify cancer-specific prognostic associations, we compared the direction of association between PRECOG meta-Z scores and our gene signatures, refining the selection of relevant cancer-specific signatures. Positive meta-Z scores show a poor prognosis, while negative scores suggest a favorable prognosis.

### 2.10 PDF-Ai-assisted evidence of involvement of signature members in the multi-optosis model

A drawback of most multi-omic studies aimed at discovering biomarkers in cancer is the lack of cross-referencing with databases. Flat lists of genes with limited features are often reported (Ravel et al., 2020; Gadepalli et al., 2021; Wang et al., 2018; Zhou and Bao, 2020), which restricts our understanding of their potential applications. We implemented a PDF generative artificial intelligence-based (PDF-Ai) strategy to provide evidence-based support for the involvement of the identified signature members. The strategy cross-references signature members with structured information from the scientific literature. This approach uses LLMs within a ChatGPT-based PDF-AI analysis tool to extract relevant data directly from the PDF corpus A, ensuring robustness and reproducibility. The method involves several key supervised, executable sequential tasks that focus on identifying mentions of gene members of the signatures, associated RCD forms, and cancer types (see Supplementary Material S1 – Methodology 4). The last step involves validating the cross-referenced data through manual review and automated checks to ensure data integrity and reliability. Any discrepancies were resolved manually to maintain the robustness of the dataset. By implementing this PDF-AI strategy, the applicability of findings is enhanced through a user-friendly data analysis tool. The structured tabular output information was compiled to create the *Cancer Regulated Cell Death Data Analyst* (https://chatgpt.com/g/g-8etzMPrtt-cancer-programmed-cell-death-data-analyst), a user-friendly, publicly accessible GPT-based chat software engineer for extracting, analyzing, and visualizing RCD data in cancer and signature members. This tool enables Chat-GPT registered users to access and interpret the relevant data efficiently, enhancing the applicability and impact of our findings.

A detailed inventory of established immunotherapy targets and their presence within the multi-omic RCD signature repertoire is presented in Supplementary Dataset S1L. The relevance and representation of these targets were assessed by cross-referencing with a curated corpus (Corpus B) of 642 manually selected PDF articles using PDF AI extraction (Supplementary Material S1).

The PDF corpora were compiled using the NCBI *pubmed* R package, and the RIS identifiers were used to download free-text using EndNote™ citation software (https://endnote.com/).

## 3 Results

**FIGURE 3:**
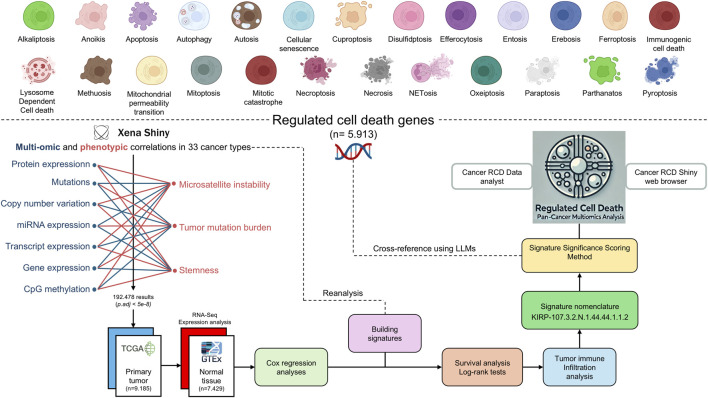
Workflow of the Multi-Optosis Model Analysis. This workflow illustrates the detailed process for constructing and analyzing a multi-optosis model focusing on 25 RCD mechanisms. The process begins with identifying 5,913 RCD-related genes using the NCBI “*Entrez”* function in R. Multi-omic and phenotypic data from TCGA Pan-Cancer are then integrated using the *“*Get Xena” R script. Expression and correlation analyses are conducted with a stringent p-value threshold (<5e-8) using the “main” R script, then consolidating all results into a single data frame. The hazard ratio is assessed for four survival metrics: overall survival (OS), disease-specific survival (DSS), disease-free interval (DFI), and progression-free interval (PFI) using Cox proportional hazards models and log-rank tests. Tumors are classified into “hot”, variable, or “cold” categories based on immune infiltration profiles. Each signature is assigned a unique nomenclature, and significance scoring is applied. The roles and involvement of gene members in various RCD forms in cancer are investigated. Finally, cross-referencing and visualization are enabled through the *CancerRCDShiny* web browser and the LLM-based *Cancer Regulated Cell Death Data Analyst* tool, allowing for interactive exploration and visualization of the findings. This structured approach integrates computational and statistical methods to enhance understanding of RCD mechanisms in cancer.

The construction and analysis of the multi-optosis model, depicted in the workflow (Figure 3), provide a comprehensive framework that integrates 25 distinct forms of RCD (Figure 1). This model is founded on a core gene set of 5,913 RCD term-based gene symbols (Supplementary Dataset S1B). The broader RCD gene inventory comprises 62,090 transcripts, spanning both primary and alternative isoforms, 882 mature miRNAs (representing both 5p and 3p strands), and 239 proteins known to be associated with cancer, including post-translational modifications. These elements form the backbone of our investigation, offering extensive coverage of RCD-related genes across cancer types.

Approximately 40% (n = 2,403) of all genes in the inventory are involved in two or more forms of RCD (Supplementary Dataset S1B). Genes exclusively associated with apoptosis account for approximately 42% (n = 2,511) of the target genes, showing no term-based association with other RCD forms. The RCD forms with the fewest associated genes are alkaliptosis, lysosome-dependent cell death, and methuosis (Supplementary Dataset S1M).

Notably, 422 genes in the inventory are established Cancer Gene Census Tier 1 driver genes (n = 584, 72.3%) in COSMIC (Catalogue Of Somatic Mutations In Cancer)^9^ (Sondka et al., 2018) and other databases (Kinnersley et al., 2024). These include oncogenes, tumor suppressors, and fusion genes, all linked to at least one RCD form (Supplementary Dataset S1B). Driver genes such as *TP53*, *AKT1*, *MTOR*, *CD274*, *PTEN*, and *STAT3* are linked to at least eight RCD forms. Among these, *TP53* stands out as the most prominent driver gene, being associated with 12 distinct forms of RCD: anoikis, apoptosis, autophagy, cellular senescence, entosis, ferroptosis, mitochondrial permeability transition, mitotic catastrophe, necroptosis, pyroptosis, necrosis, and autosis. However, several non-driver genes, such as *SIRT3*, *CXCL8*, *NFKB1*, *STING1*, and *TNF*, are noteworthy for their presence across at least eight RCD forms (Supplementary Dataset S1B).

The multi-optosis model integrates multi-omic and phenotypic reiterative correlations estimated from the TCGA Pan-Cancer secondary database (Goldman et al., 2020; Wang S. et al., 2022; Li S. et al., 2024), using R coding based on functionalities from the UCSCXenaShiny (Wang S. et al., 2022; Li S. et al., 2024). Correlation analyses were performed between the seven omic features and seven phenotypic and clinical variables in 33 cancer types. For each gene target, survival metrics were assessed using Cox proportional hazards models. Unique, single-gene, and multi-gene signatures were constructed based on feature commonalities, and their prognostic values were evaluated using the log-rank test across four survival metrics. Each signature was then queried for significant correlations with the expression profiles indicative of immune and nonimmune cell infiltrates to determine their association values with the TMC. We performed 27, 238, 756 pair associations between multi-omic, phenotypic, risk, survival and cell immune infiltration features.

The multi-omic and phenotypic features associated with each gene member in the signatures are compiled into an extensive integrative database (Supplementary Dataset S2) comprising 44,641 multi-omic signatures across 32 cancer types. None of the target genes achieved genome-wide significance with phenotypic variables in Diffuse Large B-cell Lymphoma (DLBC).

The number of elements per signature ranged from 1 to 2,052 (mean = 4.3; median = 1; Q1 = 1; Q3 = 2; P90 = 6, meaning that only 10% of signatures contain over six elements; Supplementary Dataset S2). Importantly, for the multi-member signatures, all the components share the association features, the RCD type(s), and the statistical significance level. The maximum number of member elements per omic feature is: 2,052 (Transcript), 487 (Mutation), 477 (mRNA), 423 (Methylation), 124 (CNV), 58 (miRNA) and 4 (protein) (Supplementary Figure S2).

To investigate whether the number of multi-omic signatures identified per cancer type was influenced by cohort size, we assessed the association between the number of patients and the number of signatures across the 32 tumor types analyzed. A Spearman’s rank correlation analysis revealed a positive monotonic relationship (ρ = 0.794, p = 5.9 × 10^−8^), indicating that, overall, cancer types with larger patient cohorts tended to contribute more signatures.

However, several tumor types exhibited signature-to-patient ratios that markedly exceeded the overall trend. For instance, Thymoma (THYM) yielded 1,564 signatures from only 119 patients (ratio = 13.14), Skin Cutaneous Melanoma (SKCM) produced 1,210 signatures from 102 patients (ratio = 11.86), and Kidney Chromophobe (KICH) yielded 744 signatures from 66 patients (ratio = 11.27). Even Pancreatic Adenocarcinoma (PAAD), with 178 patients, showed an elevated ratio of 10.51.

In contrast, other tumor types with substantially larger sample sizes—such as Breast Invasive Carcinoma (BRCA) with 1,092 patients—displayed a considerably lower ratio of 3.24, emphasizing that signature richness is not merely proportional to cohort size, but may reflect intrinsic biological or molecular heterogeneity across tumor types.

These results suggest that while sample size contributes to statistical power, it does not solely account for the observed variation in signature yield. Instead, intrinsic biological factors—such as tumor heterogeneity, distinct molecular programs, and RCD pathway diversity—likely shape the landscape of detectable prognostic signals.

**FIGURE 4:**
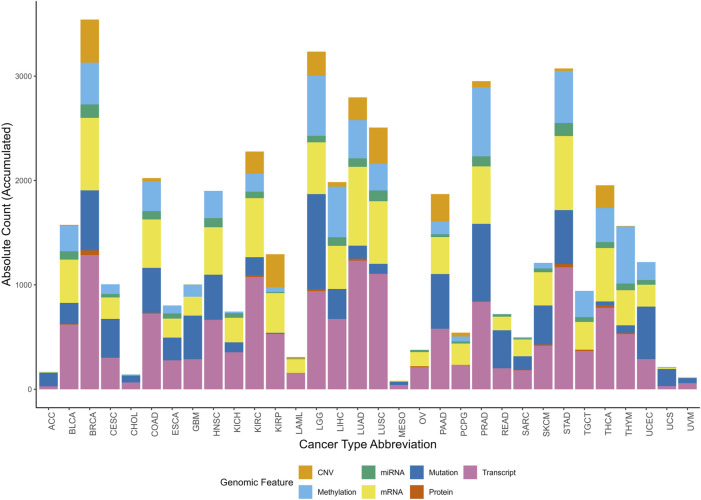
Accumulated histogram illustrating the distribution of multi-omic signatures by multi-omic feature across various cancer types. Each bar represents a unique Cancer Type Abbreviation, with colors depicting the relative proportions of signatures across multi-omic feature. The height of each bar shows the absolute accumulated count of signatures for each multi-omic feature within each cancer type. The Okabe-Ito color-blind friendly palette has been applied to enhance accessibility for all viewers.

The distribution of multi-omic signatures across omic features and cancer types is represented in Figure 4. This accumulated histogram provides insight into the proportional presence of each omic feature within different cancer types, with the absolute accumulated counts for each feature depicted.

The top-ranked cancer types, based on the number of signatures for each omic feature, reveal specific molecular patterns (Figure 4). Breast Cancer (BRCA) has the highest number of signatures associated with CNV, protein expression, transcript, and miRNA, with absolute counts of 413, 45, 1,286, and 129, respectively. Prostate Cancer (PRAD) ranks the highest in methylation-associated signatures, totaling 663, while LGG (Lower Grade Glioma) has the greatest number of mutation-linked signatures, with 910. Lung Adenocarcinoma (LUAD) exhibits a high frequency of mRNA-associated signatures, totaling 755.

Of the 5,913 target genes, 5,777 (97.7%) reached a significant correlation and are therefore included as elements in the signature database. Of the remaining genes, 101 did not achieve significance, and 35 lacked data in the Xena database. Most of the signatures include at least one apoptosis-related gene (34,500; 77.2%). This rate is expected, as 4,812 (81.4%) of the target genes are associated with apoptosis (Supplementary Dataset S2).

Among the transcript isoform signatures, the ten most frequently occurring genes were *EFEMP2*, *ABI3BP*, *TPM1*, *ELN*, *FN1*, *COL1A1*, *DCN*, *PDLIM7*, *TCF4*, and *COL1A2*, each appearing in 69–91 signatures. Collectively, these genes are associated with anoikis, apoptosis, autophagy, cellular senescence, necrosis, and pyroptosis (Supplementary Dataset S1N).

The identifier KIRP-107.3.2.N.1.44.44.1.1.2 exemplifies the nomenclature system used throughout, as shown in Figure 2. KIRP represents the cancer type abbreviation (CTAB) for Kidney Renal Papillary Cell Carcinoma, and 107 is the Gene Signature Identifier (GSI), showing the 107th signature identified for this cancer type. The Genomic Feature Code (GFC) is 3, corresponding to CNV, while the phenotypic feature contexture (PFC) is 2, indicating MSI. The Spearman Correlation Sign (SCS) is denoted as N, indicating a negative correlation. The tumor *versus* non-tumor tissue expression contexture (TNC) is 1, indicating that gene expression remains unchanged in tumor tissue compared to non-tumor tissue. The HRC is 44, based on the combination 1B2B3B4B, which shows a risk effect by all survival metrics (DSS, DFI, PFI, and OS). The survival metric contexture (SMC) is also 44, derived from the combination 1B2B3B4B, reflecting specific prognostic implications across all four survival outcomes. The tumor microenvironment contexture (TMC) is 1, indicating a correlation with an anti-tumoral environment immune profile. The tumor-infiltrating lymphocyte contexture (TIC) is 1, showing an association with “hot” profiling of immune cell infiltration. Finally, RCD is 2, signifying that the gene members are associated with two RCD forms, namely apoptosis and necrosis.

The commonalities of the signatures can be explored and analyzed purposefully or guided. Here, we exemplified the downstream analysis in two ways. The first is selecting signatures whose elements capture the highest impact rank in given omic-phenotype associations. The members of such signatures can pertain to different RCD forms (RCD Multi-Modular signatures). The second is selecting signatures that are RCD form-specific.

### 3.1 Exploring signatures with RCD multi-modular elements

Signatures composed of genes co-associated with multiple RCD forms revealed prevalent negative correlations with phenotypic traits and frequent tumor overexpression, highlighting coordinated multi-death pathway regulation.

Thirty thousand eight hundred seventy-seven signatures exhibit multi-modular involvement in RCD, where each gene component within a signature is involved in the same RCD forms. Details of these signatures are available in Supplementary Dataset S2. A negative correlation was observed between multi-omic and phenotypic features in most signatures (n = 17,069). Most multi-modular signatures were overexpressed in tumor tissues compared to non-tumor tissues (n = 13,898; Supplementary Dataset S2). Selected examples of RCD multi-modular signatures are shown in Table 1.

**TABLE 1:** Top-ranked multi-modular RCD signatures with comprehensive multi-omic representation.

Rank	Nomenclature	Signature	Elements	Omic feature	RCD count	RCD forms
48	KIRP-1086.1.3.P.3.44.34.1.1.5	P62LCKLIGAND	1	Protein	5	Apoptosis, Autophagy, Ferroptosis, Parthanatos, Autosis
48	KIRC-169.2.1.P.2.71.45.1.1.2	(CPEB4 + NF2)	2	Mutation	2	Apoptosis, Ferroptosis
47	KIRP-419.3.2.N.1.44.114.1.1.2	(SLC16A1 + SNHG3)	2	CNV	2	Apoptosis, Autophagy
47	KIRC-168.2.2.P.2.71.45.1.1.2	(CPEB4 + NF2)	2	Mutation	2	Apoptosis, Ferroptosis
47	KIRP-107.3.2.N.1.44.44.1.1.2	(CXCL10 + TNFRSF4)	2	CNV	2	Apoptosis, Necrosis
46	CESC-215.5.3.N.2.44.44.1.1.3	(ENST00000511732 + ENST00000471344 + ENST00000559488)	3	Transcript	3	Apoptosis, Autophagy, Necrosis
46	KIRP-927.3.2.N.3.15.125.1.1.3	GBP5	1	CNV	3	Apoptosis, Pyroptosis, Necrosis
45	CESC-283.6.3.N.2.44.44.1.1.3	(ITGB3 + POSTN)	2	mRNA	3	Apoptosis, Autophagy, Necrosis
44	BRCA-2207.5.3.N.3.93.95.1.1.3	ENST00000518797	1	Transcript	3	Apoptosis, Autophagy, Necrosis
44	BRCA-1496.1.3.P.3.71.71.1.1.2	CASPASE7CLEAVEDD198	1	Protein	2	Apoptosis, Autophagy
44	CESC-332.6.3.N.2.44.44.1.1.2	ADAMTS12	1	mRNA	2	Apoptosis, Necrosis
43	BRCA-1629.2.1.P.3.7.44.1.1.2	CXCR6	1	Mutation	2	Apoptosis, Autophagy
42	SKCM-711.5.3.N.3.71.71.1.1.5	ENST00000378588	1	Transcript	5	Apoptosis, Ferroptosis, NETosis, Parthanatos, Necrosis
42	CESC-420.6.3.N.2.15.44.1.1.3	COL1A1	1	mRNA	3	Apoptosis, Autophagy, Necrosis
40	HNSC-1855.4.3.P.3.71.64.1.1.4	hsa-miR-142-3p	1	miRNA	4	Apoptosis, Autophagy, Ferroptosis, Necrosis
36	PRAD-521.5.3.N.2.26.20.1.1.6	(ENST00000355622 + ENST00000394487)	2	Transcript	6	Apoptosis, Autophagy, Ferroptosis, Necroptosis, Pyroptosis, Necrosis
35	BRCA-2459.7.3.N.2.7.94.1.1.2	FHIT	1	Methylation	2	Apoptosis, Autophagy
33	BLCA-576.7.3.N.3.20.5.1.1.6	AIM2	1	Methylation	6	Apoptosis, Autophagy, Cellular senescence, Ferroptosis, Pyroptosis, Necrosis
32	LUSC-933.4.3.N.2.62.30.1.1.4	(`hsa-miR-145-3p` + “hsa-miR-145-5p”)	2	miRNA	4	Anoikis, Apoptosis, Autophagy, Necrosis
31	BRCA-1824.5.3.N.2.1.1.1.1.6	ENST00000321556	1	Transcript	6	Apoptosis, Autophagy, Cellular senescence, Ferroptosis, Pyroptosis, Mitoptosis

### 3.2 Exploring signatures with RCD-specific elements

A total of 13,764 (30.83%) signatures were identified as RCD-specific, with apoptosis-specific signatures being the most prevalent; a ranked subset revealed clinically relevant patterns across omic layers and RCD forms. These signatures encompass 20 of 25 different RCD types. Because 81.4% of genes in the inventory are term-based associated with apoptosis, we identified a large number of apoptosis-specific signatures (n = 5,793; 42%) (Supplementary Dataset S2).

We applied a sequential ranking strategy to identify the most representative signatures that prioritized both performance and comprehensive representation. For each unique RCD form, the most informative signature was selected based on the highest rank value, reflecting the overall importance of the signature. Where multiple signatures shared the same ranking value, ties were resolved by considering the highest value in additional variables in the following order: the number of gene components in the signature, TIC, TMC, SMC, and HRC. This ensured that ties were broken systematically based on biological relevance. We verified that each omic feature was included in the final selection to represent all unique omic features comprehensively. If any were missing, the highest-ranked signature for the missing omic feature was added, following the same tie-breaking hierarchy. This method allowed us to generate a ranked list of signatures that reflected their importance and ensured balanced coverage of RCD forms and multi-omic features. The top-ranked signatures by comprehensive RCD type-specific and multi-omic feature representation are presented in Table 2.

**TABLE 2:** Top-ranked RCD type-specific signatures with comprehensive multi-omic representation.

Rank	Nomenclature	Signature	Elements	Omic feature	RCD count	RCD forms
47	BRCA-70.2.1.P.2.95.47.1.2.1	(ADAMTS8 + PARP3 + UBA7)	3	Mutation	1	Apoptosis
45	BRCA-2233.5.2.N.2.95.56.1.1.1	ENST00000524317	1	Transcript	1	Apoptosis
44	KIRP-83.3.2.N.3.44.115.1.2.1	(CCNB2 + LHX2 + RPL5 + TICRR)	4	CNV	1	Autophagy
44	KIRP-408.3.2.N.2.44.126.1.2.1	(PHGDH + PRRX2)	2	CNV	1	Ferroptosis
43	CESC-69.6.3.N.2.44.44.1.1.1	(CASC15 + COL4A1 + COL4A2 + DLL4 + FAM171B+ FOXC2 + GPR4 + LAMA1 + LAMC1 + MATN3 + MSRB3 + NT5E + PXDN + RHOB + SMARCA1 + TMEM98)	16	mRNA	1	Apoptosis
42	LUAD-2334.6.1.N.2.95.95.1.2.1	NFIX	1	mRNA	1	Cellular senescence
41	BRCA-1368.6.3.P.3.44.81.1.2.1	ANLN	1	mRNA	1	Pyroptosis
39	HNSC-156.7.3.N.3.71.55.1.1.1	(CEBPE + SIRPG)	2	Methylation	1	Necrosis
38	BRCA-1503.2.1.P.2.9.39.1.2.1	CCDC178	1	Mutation	1	Anoikis
37	HNSC-656.4.3.P.3.71.71.1.2.1	(`hsa-miR-135b-3p` + “hsa-miR-135b-5p”)	2	miRNA	1	Apoptosis
36	KIRC-1057.5.3.P.2.71.71.1.2.1	ENST00000227868	1	Transcript	1	Cuproptosis
36	KIRC-1100.5.3.P.2.71.71.1.2.1	ENST00000282050	1	Transcript	1	Mitochondrial permeability transition
36	LGG-1758.5.3.P.3.95.94.2.3.1	ENST00000366898	1	Transcript	1	Mitoptosis
35	STAD-356.5.3.N.3.44.44.3.2.1	(ENST00000261037 + ENST00000463753)	2	Transcript	1	Parthanatos
34	KIRC-867.3.3.N.2.71.31.1.2.1	AJAP1	1	CNV	1	Disulfidptosis
34	KIRC-1869.6.3.N.3.35.35.1.2.1	MIIP	1	mRNA	1	Mitotic catastrophe
34	LGG-974.6.3.N.3.35.35.1.2.1	(FCGBP + NAT2)	2	mRNA	1	Necroptosis
32	LGG-1928.5.3.N.2.35.35.2.2.1	ENST00000484221	1	Transcript	1	Immunogenic cell death
30	LGG-2590.2.1.P.2.71.11.3.2.1	MTUS2	1	Mutation	1	Entosis
28	LGG-2390.7.3.P.3.71.71.2.3.1	KCNN3	1	Methylation	1	NETosis
22	THYM-1073.1.3.P.3.2.2.2.3.1	GATA3	1	Protein	1	Necrosis
22	LGG-1356.7.3.P.3.62.71.2.4.1	ATP6V0D1	1	Methylation	1	Alkaliptosis
18	PRAD-2393.7.3.P.2.0.14.3.2.1	OXSR1	1	Methylation	1	Oxeiptosis
16	THYM-573.7.3.P.3.7.2.2.4.1	ABCC11	1	Methylation	1	Efferocytosis

We next illustrate the clinical meaningfulness potential of the signature database by providing a signature for each omic feature selected from the top-ranked signatures (Table 3).

**TABLE 3:** Seven top-ranked signatures by multi-omic feature.

Rank	Nomenclature	Signature	Elements	Omic feature	RCD count	RCD forms
48	KIRC-169.2.1.P.2.71.45.1.1.2	(CPEB4 + NF2)	2	Mutation	2	Apoptosis, Ferroptosis
47	KIRP-419.3.2.N.1.44.114.1.1.2	(SLC16A1 + SNHG3)	2	CNV	2	Apoptosis, Autophagy
46	CESC-215.5.3.N.2.44.44.1.1.3	(ENST00000511732 + ENST00000471344 + ENST00000559488)	3	Transcript	3	Apoptosis, Autophagy, Necrosis
45	CESC-283.6.3.N.2.44.44.1.1.3	(ITGB3 + POSTN)	2	mRNA	3	Apoptosis, Autophagy, Necrosis
44	BRCA-1496.1.3.P.3.71.71.1.1.2	CASPASE7CLEAVEDD198	1	Protein	2	Apoptosis, Autophagy
40	HNSC-1855.4.3.P.3.71.64.1.1.4	hsa-miR-142-3p	1	miRNA	4	Apoptosis, Autophagy, Ferroptosis, Necrosis
39	HNSC-156.7.3.N.3.71.55.1.1.1	(CEBPE + SIRPG)	2	Methylation	1	Necrosis

### 3.3 mRNA-specific signatures

A total of 10,096 mRNA-specific signatures (22.6% of the dataset) were identified, many of which demonstrated significant associations with immune infiltration, transcriptional profiles, and survival risk across cancer types. These signatures (Supplementary Dataset S3) included between 1 and 477 genes per signature (mean = 3.8; median = 1; Q1 = 1; Q3 = 2; P90 = 5). Of these, 7,278 (72.1%) showed negative correlation with phenotypic features, and 6,842 (94.1%) were associated with TSM. Within this TSM-associated group, 2,479 (36.2%) signatures indicated increased risk, while 1,709 (24.9%) were protective across at least one survival metric.

Among the mRNA-specific signatures, 3,864 (38.3%) were associated with anti-tumoral transcriptional profiles, 2,101 (20.8%) with pro-tumoral profiles, and 2,750 (27.2%) with dual microenvironment profiles, reflecting diverse roles in tumor progression. Based on their correlation with immune cell infiltration profiles, the mRNA-specific signatures were categorized as “hot” (n = 273; 2.7%), showing robust immune cell presence, “cold” (n = 781; 7.7%), reflecting minimal immune infiltration, and “variable” (n = 1,540; 15.2%), denoting an intermediate or mixed immune environment.

**FIGURE 5:**
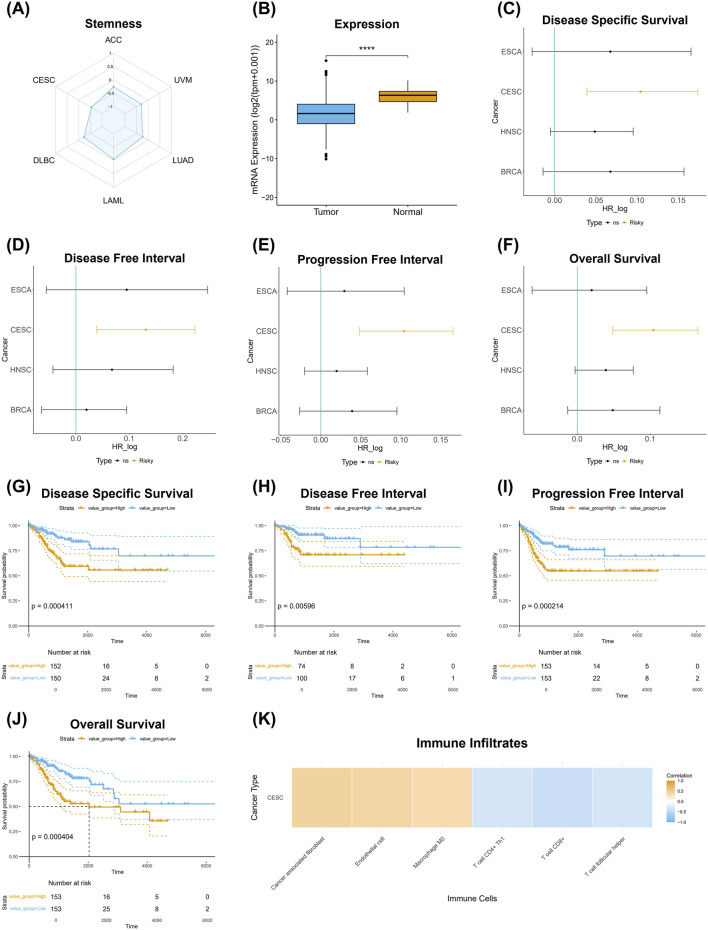
Phenotypic associations and prognostic significance of the mRNA signature CESC-283.6.3.N.2.44.44.1.1.3 in cervical squamous cell carcinoma and endocervical adenocarcinoma (CESC). **(A)** shows a radar plot illustrating the negative correlation between mRNA signature expression and TSM across multiple cancer types. **(B)** demonstrates significantly lower mRNA signature expression in tumor samples compared to normal tissue (****p < 0.0001). **(C–F)** present hazard ratio (HR) analyses evaluating the prognostic associations of the mRNA signature with clinical outcomes across various cancer types, including **(C)** Disease-Specific Survival, **(D)** Disease-Free Interval **(E)** Progression-Free Interval, and **(F)** Overall Survival, where a positive log HR indicates a risk effect of the mRNA signature. **(G–J)** display Kaplan-Meier survival curves for CESC patients stratified by high and low mRNA signature expression, with significant survival outcomes for **(G)** Disease-Specific Survival (p = 0.000411), **(H)** Disease-Free Interval (p = 0.00596), **(I)** Progression-Free Interval (p = 0.000214), and **(J)** Overall Survival (p = 0.000404). **(K)** illustrates the correlation between the mRNA signature and immune cell infiltration in CESC, highlighting associations with various immune cell types.

The identifier CESC-283.6.3.N.2.44.44.1.1.3 exemplifies an mRNA-specific signature comprising two gene members: *ITGB3* and *POSTN*, which are associated with apoptosis, autophagy, and necrosis (Table 3). These genes play diverse roles in RCD, cell survival, and migration across various cell types, contributing to cancer progression, immune modulation, and cellular stress responses. As part of the same signature, each gene consistently shares correlation signs across all phenotypic features in patients with cervical squamous cell carcinoma and endocervical adenocarcinoma (CESC) (Figure 5). Specifically, mRNA expression levels of these genes exhibit a negative correlation with TSM (Figure 5A), show lower expression in tumor samples relative to non-tumor tissue TSM (Figure 5B), and correlate with risk across all survival metrics (Figures 5C–F). Elevated expression of these genes is associated with poor prognosis across all survival metrics (Figures 5I,J). In contrast, their expression profiles correlate with an anti-tumor transcriptional profile within the tumor microenvironment and a “hot” immune infiltrate transcriptional profile (Figure 5K).

### 3.4 Transcript-level gene signatures

Transcript-level analyses revealed 16,244 signatures with widespread isoform-specific associations to stemness, prognosis, and immune context, including rare cases where all isoforms from a locus showed coordinated phenotypic correlation. Given that many gene loci express multiple transcripts through alternative splicing and promoter usage, we hypothesize that specific transcripts retain the correlation observed in the mRNA analysis. This suggests that individual transcript expression offers more precise insights into cancer progression and therapy response. By analyzing these specific transcripts, we aim to identify transcript-specific signatures that could serve as accurate prognostic and diagnostic markers, enhancing our understanding of the molecular mechanisms and heterogeneity in cancer phenotypes.

It is important to note that, for most genes, only a single transcript isoform was consistently detected at quantifiable levels across tumor samples, such that gene-level and transcript-level associations often reflect the same underlying isoform-specific signal.

We identified 16,244 transcript-specific signatures, with each signature containing between 1 and 2,052 transcript elements (mean = 5.9; median = 1; Q3 = 3 and P90 = 8) (Supplementary Dataset S4). The mean number of transcript members per signature was 3.9 (range, 1–49) for signatures associated with risk and 4.1 (range, 1–76) for those associated with protection (Supplementary Dataset S4). Approximately 62.8% (n = 10,207) were associated with risk or protection in at least one survival metric. From those, we identified 605 (5.9%) signatures associated with risk across all patient survival metrics and 270 (2.7%) signatures with protective association in all four patient survival metrics. Most signatures ascribed correlations between transcript expression and TSM (86% for risk and 92% for protective signatures). Transcript signature overexpression was the feature most frequently associated with risk (54.5%), whereas underexpression was mainly associated with protection (47%). Example: CESC-215.5.3.N.2.44.44.1.1.3 refers to the transcript expression (ENST00000511732 + ENST00000471344 + ENST00000559488), which negatively correlated with stemness in CESC patients (Supplementary Figure S3A). There was significantly lower transcript signature expression in tumor samples compared to normal tissue (Supplementary Figure S3B). Transcript overexpression is associated with increased risk in DSS (Supplementary Figure S3C), DFI (Supplementary Figure S3D), PFI (Supplementary Figure S3E), and OS (Supplementary Figure S3F) survival metrics. Transcript signature overexpression was associated with poor prognosis in all survival metrics (Supplementary Figure S3G–J). Transcript signature expression correlated with an anti-tumor transcriptional profile within the tumor microenvironment and a “hot” immune infiltrate transcriptional profile (Figure 3K).

**FIGURE 6:**
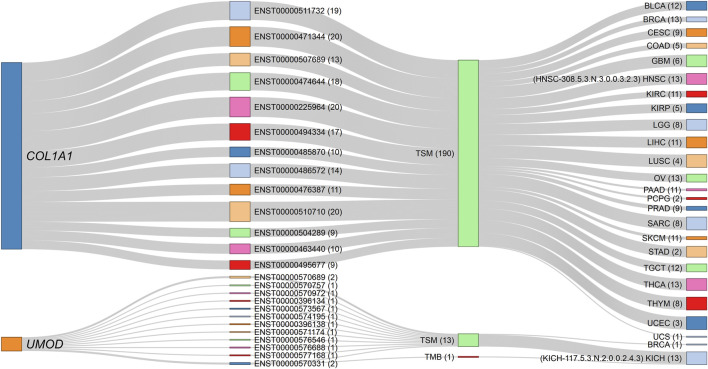
Sankey diagram depicting the negative correlations of *COL1A1* and *UMOD* gene isoforms with stemness across specific cancer signatures. The source nodes represent the *COL1A1* and *UMOD* gene loci, each linked to their respective transcript isoforms identified in the dataset. The numbers in parentheses indicate the number of connection strokes. All 13 *COL1A1* isoforms consistently exhibit negative associations with TSM in the HNSC-308.5.3.N.3.0.0.3.2.3 signature (comprising 46 elements), while all 12 *UMOD* isoforms similarly show negative correlations with TSM in the KICH-117.5.3.N.2.0.0.2.4.3 signature (comprising 61 elements). The thickness of the stroke connection lines represents the frequency of correlations between nodes (genes, transcripts, phenotypes, and cancer types), emphasizing the uniform contribution of each gene’s isoforms to the observed phenotype. This consistent transcript-level correlation across all isoforms of *COL1A1* and *UMOD* suggests a coordinated regulatory function in modulating TSM within these cancer contexts. The corresponding dynamic network diagram is available in Supplementary Material S1 (Supplementary Figure S12).

An interesting observation in multi-transcript genes is worth noting; first, the highest number of transcripts per gene that correlated with a phenotype in a cancer type was 19, and was limited to the *CD36* (19 out of 24 transcripts), *ABI3BP* (19/29), and *TCF4* (19/93) genes. Second, correlations with all transcript isoforms per gene were extremely rare. Examples include *COL1A1* (a known cancer driver gene) with its 13 isoforms, which are negatively correlated with stemness in the multi-element signature HNSC-308.5.3.N.3.0.0.3.2.3, comprising 46 member elements, and *UMOD* with its 12 transcripts, also negatively associated with stemness in the multi-element signature KICH-117.5.3.N.2.0.0.2.4.3, which comprises 61 member elements (Figure 6). Thus, for those signatures, all the *COL1A1-and UMOD-*specific transcripts consistently retained the correlation with stemness. Hence, for these genes, the entire gene loci, rather than individual isoforms, uniformly contribute to the observed phenotype, highlighting a coordinated regulatory role of these genes in maintaining the correlation with stemness. The uniformity across all isoforms within a gene is an uncommon and significant finding, underscoring the comprehensive influence of these genes on the stemness phenotype.

**FIGURE 7:**
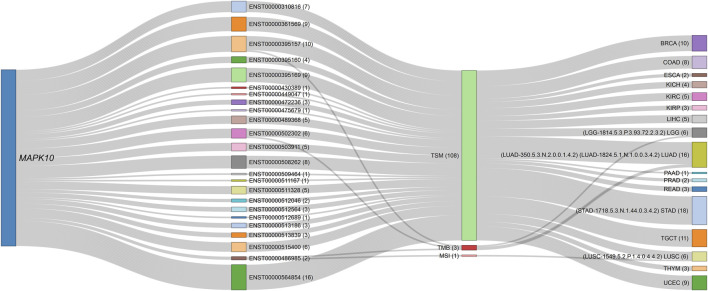
Sankey diagram illustrating transcript-specific associations of the *MAPK10* gene across various phenotypes and cancer types. The *MAPK10* gene locus appears as the source node, connected to its specific transcript isoforms identified in the dataset. Each transcript is further linked to phenotypic profiles (i.e., TSM, TMB, MSI) and mapped to cancer types such as BRCA, COAD, and GBM. The numbers in parentheses indicate the number of connection strokes. The thickness of each link represents the frequency of correlation between *MAPK10* transcripts and the respective phenotypes or cancer types, highlighting both transcript-specific and phenotype-driven associations within multi-transcript gene correlations. For example, the transcript ENST00000486985 (signature identifier: LUSC-1549.5.2.P.1.4.0.4.4.2) shows a positive correlation with MSI in patients with LUSC, while the isoform ENST00000502302 (LUAD-1824.5.1.N.1.0.0.3.4.2) demonstrates a negative correlation with TMB in LUAD patients. The corresponding interactive proportional node dynamic network is available in Supplementary Material S1 (Supplementary Figure S13).

In contrast, for most multi-transcript RCD genes, the correlations were transcript isoform-specific rather than involving the entire gene locus transcript repertoire. Isoform-specific signatures refer to the unique associations of transcript variants from a single gene locus with distinct clinical and phenotypic outcomes. These signatures enable the identification of specific transcript variants that contribute to cancer progression, prognosis, and therapeutic response. Specifically, for the *MAPK10* gene, which has 192 known transcripts, our analysis revealed that only 24 transcripts showed significant correlations with metrics such as TSM, TMB, or MSI across 17 cancer types, appearing in up to 47 different signature identifiers (Figure 7, Supplementary Dataset S1O). The remaining 180 transcripts from this locus showed no meaningful association. The highest number of *MAPK10* transcript members per signature was 12, observed in LUAD-350.5.3.N.2.0.0.1.4.2. Notably, distinct *MAPK10* transcript isoforms were associated with divergent phenotypes across cancer types. For example, ENST00000486985 expression was positively correlated with MSI in lung squamous cell carcinoma (LUSC) patients (LUSC-1549.5.2.P.1.4.0.4.4.2). In contrast, ENST00000502302 was negatively correlated with TMB in lung adenocarcinoma (LUAD) patients (LUAD-1824.5.1.N.1.0.0.3.4.2). Similarly, ENST00000395169 exhibited a protective role correlating with favorable outcomes in LGG (LGG-1814.5.3.P.3.93.72.2.3.2), whereas ENST00000395160, a different isoform from the same locus, was associated with risk, by four survival metrics, in stomach adenocarcinoma (STAD-1718.5.3.N.1.44.0.3.4.2). These isoform-specific correlations underscore the heterogeneity within the *MAPK10* gene locus, where distinct transcripts contribute variably to cancer progression, phenotypic features, and therapeutic responses across cancer types.

Table 4 summarizes the transcript-specific correlations of the *MAPK10* gene with cancer types, phenotypic characteristics, and prognostic outcomes, as detailed above. Each transcript is linked to a unique signature identifier, highlighting its distinct role in cancer progression, its associated phenotypic features, and therapeutic relevance.

**TABLE 4:** Examples of transcript-specific correlations of *MAPK10* with cancer types, phenotypic features, and prognostic outcomes.

Signature identifier	Transcript ID	Cancer type	Phenotypic correlation	Correlation direction	Comment
LGG-1814.5.3.P.3.93.72.2.3.2	ENST00000395169	LGG (Lower-Grade Glioma)	Favorable outcomes	Protective	Correlated with better survival outcomes
STAD-1718.5.3.N.1.44.0.3.4.2	ENST00000395160	STAD (Stomach Adenocarcinoma)	Poor prognosis	Risk	Linked to worse survival outcomes
LUSC-1549.5.2.P.1.4.0.4.4.2	ENST00000486985	LUSC (Lung Squamous Cell Carcinoma)	MSI	Positive	Transcript positively correlated with MSI phenotype
LUAD-1824.5.1.N.1.0.0.3.4.2	ENST00000502302	LUAD (Lung Adenocarcinoma)	TMB	Negative	Transcript negatively correlated with high TMB, a hallmark of poor prognosis in LUAD.

### 3.5 miRNA-specific signatures

A total of 1,470 miRNA-specific signatures were identified, with over half associated with prognostic outcomes and immune phenotypes, revealing transcriptomic roles for miRNAs such as hsa-miR-142-3p across multiple RCD forms and cancer types. The miRNA-specific signatures are composed of 1–58 elements (mean = 2.2; median = 1; Q3 = 2; P90 = 4). Of these, 954 (64.9%) contain a single miRNA element. Among the miRNA-specific signatures, 786 (53.5%) correlated with risk or protection in at least one survival metric. Of these, 41 (5.2%) correlated with risk and 16 (2%) with protection in all four metrics of survival (Supplementary Dataset S5). The miRNA signatures correlated with distinct tumor microenvironment profiles, including anti-tumoral, pro-tumoral, and variable conditions. We highlight the signature HNSC-1855.4.3.P.3.71.64.1.1.4, which corresponds to hsa-miR-142-3p, the mature form of *MIR142* in head and neck squamous cell carcinoma (HNSC) patients*.* Cross-referencing public datasets revealed *MIR142* is involved in four RCD forms—apoptosis, autophagy, ferroptosis and necrosis—emphasizing its critical role in hematopoiesis, immune regulation, and cancer progression by modulating various target genes involved in T cell differentiation, inflammation, and tumorigenesis.

hsa-miR-142-3p expression shows a positive correlation with TSM (Supplementary Figure S4A). It is overexpressed in HNSC tumors as compared with non-tumor tissues (Supplementary Figure S4B). While hsa-miR-142-3p overexpression was associated with protection in DSS (Supplementary Figure S4C), PFI (Supplementary Figure S4E) and OS (Supplementary Figure S4F), the underexpression was associated with poorer prognosis, as reflected in DSS (Supplementary Figure S4G), PFI (Supplementary Figure S4I), and OS (Supplementary Figure S4J). Furthermore, hsa-miR-142-3p expression was linked to an anti-tumoral profile in the tumor microenvironment, characterized by a “hot” immune infiltrate, indicative of active immune engagement (Supplementary Figure S4K).

### 3.6 Gene-specific CpG methylation signatures

We identified 6,109 CpG methylation-specific gene signatures, most of which were associated with TSM and included subsets linked to immune infiltration profiles and patient outcomes across all survival metrics. The gene-specific CpG methylation signatures exhibit element counts ranging from 1 to 423 per signature (mean = 3.2; median = 1; Q1 = 1; Q3 = 2; P90 = 5), of which 4,246 (69.5%) contain a single CpG Methylation-specific member. The majority (n = 5,350; 87.6%) was associated with TSM. Of these, 192 (3.59%) were linked to an increased risk, while 60 (1.12%) were protective in all four metrics of survival (Supplementary Dataset S6). These signatures were further stratified based on their correlation with tumor microenvironment profiles, showing anti-tumoral (n = 98; 38.9%), pro-tumoral (n = 42; 16.7%), and dual (n = 54; 21.4%) characteristics. The methylation signatures associated with TSM were classified according to their association with immune cell infiltration profiles, showing “hot” (n = 6; 2.4%), “cold” (n = 16; 6.4%), or variable (n = 25; 9.9%) immune phenotypes.

For instance, the signature HNSC-156.7.3.N.3.71.55.1.1.1 demonstrates a negative correlation between CpG methylation at the *CEBPE* and *SIRPG* loci and TSM in HNSC patients (Supplementary Figure S5A). *CEBPE* and *SIRPG* mRNA expression levels were higher in tumor than in non-tumor samples (Supplementary Figure S5B). *CEBPE* and *SIRPG* mRNA expression levels are associated with protection in DSS (Supplementary Figure S5C), PFI (Supplementary Figure S5E), and OS (Supplementary Figure S5F). High methylation levels at *CEBPE* and *SIRPG* are linked to a poorer prognosis in all survival metrics (Supplementary Figures S5G–J). Furthermore, *CEBPE* and *SIRPG* mRNA expression correlates with an anti-tumor microenvironment transcriptional profile and is linked to a “hot” immune infiltration profile in HNSC patients (Supplementary Figure S5K).

### 3.7 Protein-specific signatures

We identified 258 protein-specific signatures, predominantly correlated with TSM and microenvironmental phenotypes, including a small subset linked to survival outcomes. The protein-specific signatures contain between 1 and 4 elements (mean = 1.1; median = 1; Q1 = 1; Q3 = 1; P90 = 1). Of these, the majority (254; 98.5%) exhibited a correlation with TSM, with 153 (60.2%) showing a positive correlation and 101 (39.8%) displaying a negative correlation. Among these, 7 (2.76%) were associated with an increased risk, while 1 (0.4%) was linked to protective effects in all four metrics of survival (Supplementary Dataset S7). Furthermore, 47 (18.22%) protein-specific signatures correlated with anti-tumoral profiles and 147 (57%) with dual tumor microenvironment profiles. Protein signatures also correlated with immune phenotypes categorized as “hot” (4; 1.3%), “cold” (11; 4.26%), or “variable” (17; 6.6%). Example: BRCA-1496.1.3.P.3.71.71.1.1.2 refers to the expression of the CASPASE7CLEAVEDD198 protein modification, which positively correlated with stemness in BRCA patients (Supplementary Figure S6A). There was significantly higher mRNA expression for the gene encoding the signature element in tumor samples compared to normal tissue (Supplementary Figure S6B).

Protein overexpression is protective in DSS (Supplementary Figure S6C), PFI (Supplementary Figure S6E) and OS (Supplementary Figure S6F), survival metrics. Low protein expression was associated with poor prognosis in the same survival metrics (Supplementary Figures S6G,I,J). Moreover, CASPASE7CLEAVEDD198 expression correlated with anti-tumoral microenvironment and “hot” immune infiltration profiles (Supplementary Figure S6K).

### 3.8 Mutation-specific signatures

We identified 8,022 mutation-specific signatures, predominantly associated with TMB and immunophenotypic heterogeneity, with a minority showing prognostic correlations. The signatures comprise 1 to 487 elements (mean = 3.6; median = 1; Q1 = 1; Q3 = 1; P90 = 5). Of these, 5,464 (68.1%) consisted of a single element. The majority showed a positive correlation with TMB (5,880; 73.3%) and MSI (2,136; 26.6%), while a small subset (3; 0.04%) showed a positive correlation with TSM (Supplementary Dataset S8).

The TMB-associated signatures were linked to risk (n = 229; 3.9%), protection (n = 96; 1.63%), “cold” immune cell profiles (n = 437; 5.45%), “hot” immune profiles (n = 200; 3.4%), “variable” immune profiles (n = 627; 10.7%), pro-tumoral (n = 687; 11.7%), anti-tumoral (1,426; 24.3%) and dual tumor microenvironment profiles (n = 1,501; 25.5%) (Supplementary Dataset S8). For example, signature KIRC-169.2.1.P.2.71.45.1.1.2, which features the mutation commonalities of *CPEB4* and *NF2* genes, is positively associated with TMB in KIRC patients (Supplementary Figure S7A). mRNA expression of the signature element was significantly higher in tumor *versus* non-tumor samples (Supplementary Figure S7B). mRNA expression of those genes was a protective factor in DSS (Supplementary Figure S7C), PFI (Supplementary Figure S7E) and OS (Supplementary Figure S7F). Mutations in those genes were associated with poor prognosis in all metrics (Supplementary Figures S3G–J). mRNA expression of the signature elements correlated with anti-tumoral microenvironment and “hot” immune infiltration profiles (Supplementary Figure S7K).

### 3.9 CNV-specific signatures

We identified 2,442 CNV-specific signatures, over half of which were associated with TSM, with a minority demonstrating consistent prognostic and immune microenvironment correlations. Each CNV-specific signature comprises 1 and 124 elements (mean = 2.4; median = 1; Q1 = 1; Q3 = 2; P90 = 4), 675 (27.6%) of which comprise >1 element. Most of the CNV-specific signatures (1,313; 53.8%) were associated with TSM (Supplementary Dataset S9). Among these, 915 (69.9%) exhibited a negative correlation, while 398 (30.3%) demonstrated a positive correlation. Among the CNV signatures that correlated with TSM, 54 (4.1%) were associated with risk or protection across all four survival metrics. A portion of these signatures correlated with anti-tumoral (n = 24; 44.4%), pro-tumoral (n = 8; 14.8%) and dual expression profiles (n = 19; 35.2%). These signatures were associated with tumor immune infiltration, characterized as “cold” (n = 11; 20.4%), “hot” (n = 3; 5.6%) or “variable” (n = 9; 16.7%).

For example, signature KIRP-107.3.2.N.1.44.44.1.1.2, comprising *CXCL10* and *TNFRSF4*, showed CNV negatively correlated with MSI in KIRP Supplementary Figure S8A). mRNA expression of the CNV signature constituents was unchanged between tumors and non-tumor samples (Supplementary Figure S8B). mRNA overexpression of these genes was associated with an increased risk in DSS (Supplementary Figure S8C), DFI (Supplementary Figure S8D), PFI (Supplementary Figure S8E) and OS (Supplementary Figure S8F). Patients with CNV deletions exhibited poor prognosis across all survival metrics (Supplementary Figures S8G–J). Furthermore, mRNA expression of *CXCL10* and *TNFRSF4* was associated with anti-tumoral and “hot” immune microenvironment profiles (Supplementary Figure S8K).

Table 5 provides a consolidated overview of the classification and distribution of multi-omic signatures, including mRNA, transcript, miRNA, CpG methylation, CNV, mutation, and protein, according to their hazard-risk assessment (risky, protective, or poor prognostic signatures) and their correlation with tumor microenvironment and immune phenotype profiles. These profiles are further categorized based on anti-tumoral, pro-tumoral, or dual microenvironment classifications, as well as immune phenotypes, into “hot,” “cold,” or variable categories. This summary highlights the complexity of prognostic and therapeutic insights derived from distinct multi-omic features, providing a deeper understanding of their contextual relevance in cancer research and facilitating the discovery of new biomarkers and therapeutic targets for enhanced patient outcomes. By integrating diverse molecular features, we highlight the differential associations of multi-omic signatures with tumor prognosis and therapeutic informativeness, defined as the clinical relevance of biomarkers in guiding treatment decisions.

**TABLE 5:** Classification and distribution of multi-omic signatures by clinical outcomes, hazard ratio, and therapeutic informativeness.

Omic feature	Hazard ratio									Kaplan-meier				Therapeutic informativeness					
	Total	Risky				Protective				Poorer prognosis				TME			TIC		
		DSS	DFI	PFI	OS	DSS	DFI	PFI	OS	DSS	DFI	PFI	OS	Anti-tumoral	Pro-tumoral	Dual	Hot	Cold	Variable
mRNA	10,096	2,372	1,136	2,207	2,400	1,414	728	1,344	1,151	2,734	1,405	2,630	2,733	3,864	2,101	2,750	273	781	1,540
Transcript	16,244	3,737	1987	3,527	3,724	2,291	1,393	2,231	1968	4,485	2,535	4,352	4,428	4,176	4,222	6,360	368	968	2006
miRNA	1,470	291	121	249	248	190	98	161	185	328	165	301	310	572	175	537	35	37	141
Mutation	8,022	1900	957	1850	1802	987	558	1,106	1,022	722	312	1,165	679	2003	1,000	2,175	251	437	913
CNV	2,442	591	323	519	562	371	160	350	259	879	492	827	717	724	316	746	74	148	403
Methylation	6,109	1,255	605	1,241	1,289	730	410	722	618	1,211	753	1,327	1,460	1,632	850	1708	197	419	711
Protein	258	37	14	39	37	18	4	22	27	43	20	46	49	47	0	147	4	11	17
Total	44,641	10,183	5,143	9,632	10,062	6,001	3,351	5,936	5,230	10,402	5,682	10,648	10,376	13,018	8,664	14,423	1,202	2,801	5,731

### 3.10 Clinically meaningful signatures

By integrating diverse molecular features, we identified 167 clinically meaningful signatures across five omic features: Transcript, mRNA, CNV, Methylation, and Mutation. These signatures are characterized by consistent associations with prognostic outcomes and immune microenvironment phenotypes in 11 cancer types, including STAD, PRAD, LUSC, LUAD, LGG, KIRP, KIRC, HNSC, CESC, BRCA, and ACC (Supplementary Dataset S1P). The selection process focused on signatures with significant associations with hazard ratio and prognostic metrics across all four survival outcomes: DSS, DFI, PFI and OS. These signatures also showed robust correlations with immune infiltration profiles, which were categorized into anti-tumoral, pro-tumoral, or dual microenvironment roles, and immune phenotypes classified as “hot,” “cold,” or “variable”. Among these, the top most clinically significant signatures are presented in Table 6.

**TABLE 6:** Top most clinically meaningful signatures.

Rank	Nomenclature	Signature	Omic feature	RCD form	HRC				SMC				TMC	TIC
					DSS	DFI	PFI	OS	DSS	DFI	PFI	OS		
47	KIRP-419.3.2.N.1.44.114.1.1.2	(SLC16A1 + SNHG3)	CNV	Apoptosis, Autophagy	Deleted, Duplicated	Deleted	Deleted	Deleted, Duplicated	Risky	Risky	Risky	Risky	anti-tumoral	Hot
47	KIRP-107.3.2.N.1.44.44.1.1.2	(CXCL10 + TNFRSF4)	CNV	Apoptosis, Necrosis	Deleted	Deleted	Deleted	Deleted	Risky	Risky	Risky	Risky	anti-tumoral	Hot
46	CESC-215.5.3.N.2.44.44.1.1.3	(ENST00000511732 + ENST00000471344 + ENST00000559488)	Transcript	Apoptosis, Autophagy, Necrosis	High	High	High	High	Risky	Risky	Risky	Risky	anti-tumoral	Hot
45	CESC-283.6.3.N.2.44.44.1.1.3	(ITGB3 + POSTN)	mRNA	Apoptosis, Autophagy, Necrosis	High	High	High	High	Risky	Risky	Risky	Risky	anti-tumoral	Hot
45	BRCA-2233.5.2.N.2.95.56.1.1.1	ENST00000524317	Transcript	Apoptosis	High	Low	High	Low	Protective	Protective	Protective	Protective	anti-tumoral	Hot
44	CESC-332.6.3.N.2.44.44.1.1.2	ADAMTS12	mRNA	Apoptosis, Necrosis	High	High	High	High	Risky	Risky	Risky	Risky	anti-tumoral	Hot
44	CESC-143.5.3.N.2.44.44.1.1.1	(ENST00000299402 + ENST00000606197 + ENST00000341529 + ENST00000375820 + ENST00000360467 + ENST00000249749 + ENST00000304698 + ENST00000323040 + ENST00000389658 + ENST00000488064 + ENST00000258341 + ENST00000407540 + ENST00000341712 + ENST00000303375 + ENST00000299964 + ENST00000546313 + ENST00000535401 + ENST00000342694 + ENST00000257770 + ENST00000355841 + ENST00000356435 + ENST00000620489 + ENST00000272233 + ENST00000369515 + ENST00000505615)	Transcript	Apoptosis	High	High	High	High	Risky	Risky	Risky	Risky	anti-tumoral	Hot
44	BRCA-693.5.3.N.2.95.95.1.1.1	(ENST00000445609 + ENST00000356495 + ENST00000414546 + ENST00000528349 + ENST00000600277 + ENST00000259075 + ENST00000474710 + ENST00000557983)	Transcript	Necrosis	Low	Low	Low	Low	Protective	Protective	Protective	Protective	anti-tumoral	Hot
43	CESC-69.6.3.N.2.44.44.1.1.1	(CASC15 + COL4A1 + COL4A2 + DLL4 + FAM171B+ FOXC2 + GPR4 + LAMA1 + LAMC1 + MATN3 + MSRB3 + NT5E + PXDN + RHOB + SMARCA1 + TMEM98)	mRNA	Apoptosis	High	High	High	High	Risky	Risky	Risky	Risky	anti-tumoral	Hot

### 3.11 Identification of potential therapeutic targets through known drug-gene interactions in top-ranked gene signatures

To identify potential therapeutic targets, we analyzed the gene components of the leading multi-modular signatures (Table 1), RCD type-specific signatures (Table 2), multi-omic feature signatures (Table 3) and top clinically meaningful signatures (Table 6). Collectively, these top 45 signatures (Supplementary Dataset S1Q) encompass 84 distinct genes (Supplementary Dataset S1R). By inputting this list into the DGIdb (Cannon et al., 2024), we found that 27 of the 84 genes are associated with 146 known drug interactions, as detailed in Supplementary Dataset S1S. Notably, 59.6% (n = 87) of these interactions involve drug inhibitors. The genes with the highest number of drug interactions include *APBB1*, *NAT2*, *ITGB3*, *RHOB*, *TLR4*, *ATP5F1A*, *TNFRSF4*, *GATA3*, *PARP3*, *RPL5* (Supplementary Figure S11).

### 3.12 Independent validation of prognostic signatures using PRECOG

**FIGURE 8:**
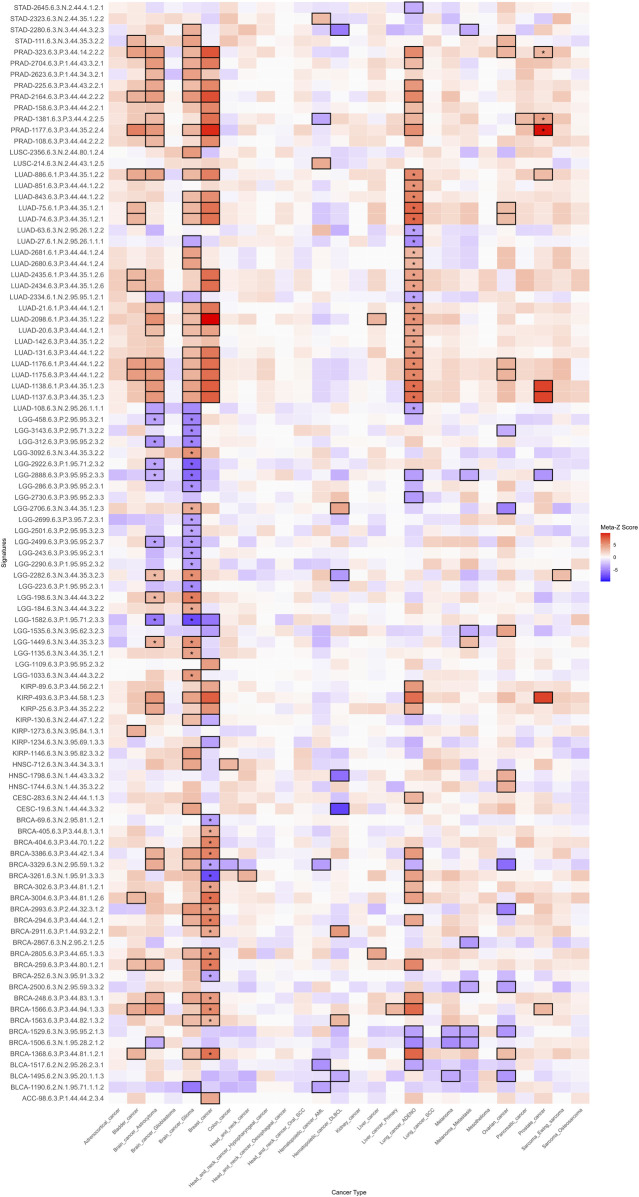
Heatmap of prognostic meta-Z scores from the PRECOG independent database. This heatmap illustrates the association between mRNA-specific signatures (y-axis) and PRECOG cancer types (x-axis). Median meta-Z scores were computed based on overall survival (OS) metrics. Cells are color-coded: blue denotes a favorable prognosis (negative meta-Z scores), red indicates a poor prognosis (positive meta-Z scores), and gray represents neutral or non-significant values. Black-bordered cells highlight statistically significant associations (|Meta-Z| > 3.09 or < −3.09, p < 0.001). The asterisk within the black-bordered cells marks signatures whose prognostic values were validated in both direction and strength in TCGA-equivalent PRECOG cancer types.

To ensure the robustness and generalizability of our findings, we assessed the prognostic value of 126 top, clinically meaningful, mRNA-specific signatures (Supplementary Dataset S1K) using the independent PRECOG database. Of the 126 signatures selected for their association with risk, protection, and poor prognosis—as well as their links to anti-tumoral, pro-tumoral, or dual microenvironment cell profiles and immune infiltrates—we successfully validated 73 signatures in five PRECOG cancer types (Lung cancer ADENO, Breast cancer, Brain cancer Glioma, Brain cancer Astrocytoma, and Prostate cancer). These PRECOG cancer types are equivalent to the TCGA cancer types LUAD, BRCA, LGG, and PRAD, in which these signatures were initially identified (Figure 8). This validation underscores the clinical relevance of these signatures and their potential utility in diverse patient populations.

### 3.13 *CancerRCDShiny*: exploring multi-omic signatures in RCD for cancer research

We implemented *CancerRCDShiny* (https://cancerrcdshiny.shinyapps.io/cancerrcdshiny/), a tool designed to facilitate the exploration and analysis of signatures associated with RCD forms in cancer. This R Shiny app is tailored for researchers and clinicians aiming to uncover the molecular underpinnings of cancer through the lens of cell death processes. *CancerRCDShiny* integrates a robust database encompassing 25 distinct RCD forms and 32 cancer types, enabling users to explore the intricate relationships between signatures and cancer phenotypes. The app employs rigorous genome-wide significance filters to identify the most relevant signatures, ensuring access to high-confidence data for thorough analysis and interpretation. Users can explore multiple gene features and phenotypic attributes, providing a comprehensive view of the genetic landscape associated with RCD in cancer. The app features a user-friendly interface with dynamic visualization tools, enabling users to easily navigate data, create custom plots, and generate detailed reports. Researchers can tailor their queries to specific RCD forms, cancer types, or omic features, facilitating targeted investigations. *CancerRCDShiny* is an essential resource for precision oncology, empowering researchers to uncover novel insights and advance cancer research.

*CancerRCDShiny* also contains an *RCD Multi-omic Signature Identifier Interpreter* function that deciphers the complex nomenclature of the signatures. This function enables users to paste any RCD signature identifier from the database and download the interpreted identifier in text format.

### 3.14 Performance of the cancer regulated cell death data analyst tool

The Cancer Regulated Cell Death Data Analyst is a specialized GPT-based software tool designed to extract and process information from various file formats, generating structured tabular outputs in. csv format to address specific research queries related to RCD in cancer. It offers robust capabilities, including automated data cleaning, integration with external databases, and NLP techniques for extracting insights from unstructured text. The tool supports interactive dashboards for real-time visualization, functional annotation and enrichment analysis, predictive modeling using machine learning, and customizable reporting. Additional features include secure user authentication, data encryption, API access for seamless integration with other software tools, collaborative functionalities for team-based analysis, version control for data and workflows, and educational resources. It also provides advanced R code suggestions for in-depth analysis, such as data visualization through plots and images, explicitly tailored for RCD research. A built-in feedback mechanism ensures continuous improvement, while enhanced plotting and imaging capabilities further refine data interpretation and analysis. The tool can be accessed at URL: https://chatgpt.com/g/g-8etzMPrtt-cancer-regulated-cell-death-data-analyst.

## 4 Discussion

### 4.1 Holistic approach and context-specific analysis

Our multi-optosis model is integrative and holistic, querying 5,913 genes associated with RCD, encompassing 62,090 transcripts, 882 mature miRNAs and 239 cancer-associated proteins and protein modifications (for 193 genes) from 25 distinct RCD forms. The model assumes non-uniformity in the activity and effects of the RCD gene components across different cancer types. Each cancer type is analyzed separately, ensuring that the unique biological contexts and specific molecular mechanisms of each cancer type are thoroughly considered. When querying target genes, we treat the RCD gene inventory as a whole; however, each gene is conceptually tagged to one or more RCD forms. This approach enables us to account for the unique biological contexts and specific molecular mechanisms of each cancer type, thereby ensuring a comprehensive understanding of the associations between RCD gene partners in cancer progression and treatment resistance. By combining these elements, our model uncovers new biomarkers and therapeutic target candidates, opening avenues for more effective cancer treatments.

The signature database developed in this study offers a valuable resource for advancing cancer research and treatment through multiple RCD signaling pathways. The signature identifiers are enriched with meaningful information encoded in the nomenclature system, unveiling hidden correlations between multi-omic and phenotypic features. Our rank-scoring system integrates multiple critical factors to assess the overall significance and correlation of each signature, providing preliminary evidence for their prognostic value. This method offers a comprehensive framework for evaluating signatures in cancer research. Our process ensures a balanced and accurate assessment of each signature’s relevance by considering multiple factors, including cancer type, survival metrics, and multi-omic and phenotypic features. The scoring of signatures can facilitate the prioritization of signatures for further investigation, ultimately accelerating the discovery of actionable insights and improving patient outcomes.

Currently, no signature identifier system in the literature incorporates multi-omics features as comprehensively as our model does. Most studies on signatures related to RCD and cancer list signatures based on a single type of association (Wang and Zhang, 2024; Bao et al., 2014; Cai et al., 2020; Chen et al., 2020; Chang et al., 2021; Modarres et al., 2021; Wan et al., 2021; Bian et al., 2022; Li et al., 2022; Yao et al., 2022; Chen L. et al., 2023; Li J. et al., 2024; Zhou X. et al., 2024). Our model’s novel integration of multi-omics features and multiple phenotypic attributes provides a more holistic and informative framework for understanding cancer biology. The uniqueness of our integrated multi-omic, multi-feature signature discovery approach significantly enhances the potential of key biomarkers and therapeutic targets.

However, we recognize the necessity of thorough validation across independent datasets to confirm the reliability of these signatures in clinical settings. Therefore, validation in independent datasets is necessary to establish the clinical applicability of our findings fully.

As a discovery-phase analysis encompassing over 44,000 mono-omic signatures, our model prioritized genome-scale screening using univariate Cox models. While this approach enables broad comparability across tumor types and omic layers, we recognize that downstream validation through multivariate modeling—including adjustment for clinical confounders such as age, sex, and stage—is essential to confirm independent prognostic utility. Importantly, implementation of covariate-adjusted survival modeling would entail redefinition of the signature construction logic to ensure that association signals are preserved across clinical strata, and would require computational infrastructure beyond the current framework. We plan to address this in future validation phases.

The ability to stratify tumors or patients based on multiple layers of omic regulation (e.g., miRNA and methylation) is particularly valuable for building interpretable prognostic and mechanistic models. We recognize that resampling-based methods, such as bootstrapping or subsampling, are valuable tools for assessing the stability of candidate signatures. Given the exploratory and genome-wide scope of the present analysis—encompassing over 44,000 multi-omic signatures across 33 cancer types—bootstrap-based stability testing was not implemented at this stage due to computational limitations. In this discovery context, the presented top-ranked signatures are demonstrative in nature, selected to illustrate biologically and clinically meaningful associations across omic layers. They are not intended as definitive biomarkers. Systematic prioritization and validation of signature robustness will be the focus of future follow-up analyses.

### 4.2 Interpretation of multi-omic and phenotype correlations and their signs

The signatures identified through specific multi-omic and phenotype correlations are candidate proxies for diagnosis, prognosis, or therapeutic response. Integrating positive and negative correlations into our analysis provides a more comprehensive understanding of the signatures associated with various cancer phenotypes. This thorough approach enables the identification of potential oncogenes and tumor suppressors, paving the way for the development of more tailored and effective cancer treatments. For instance, positive correlations between gene expression and TSM could indicate aggressive cancer phenotypes, metastasis, and therapy resistance. Examples include overexpression of the pluripotency- and apoptosis-related *POU5F1* (*OCT4*) (i.e., TGCT-15.6.3.P.3.0.0.2.4.2), *SOX2* (i.e., LUSC-2293.6.3.P.3.62.30.1.4.3), and *NANOG* (i.e., TGCT-4.6.3.P.3.0.0.2.4.1) genes in various cancer types associated with stemness and poor prognosis (Clemente-Perivan et al., 2020; Chiou et al., 2010; Wu et al., 2012; Gutekunst et al., 2013; Upadhyay et al., 2020; Mehrzad et al., 2022; Fang et al., 2023; von Eyben et al., 2023; Zhu and Xu, 2024).

Negative correlations provide equally critical insights. A negative correlation between gene CNV and a particular phenotype (i.e., stemness) could indicate genes that suppress aggressive traits or resistance mechanisms. For example, *TP53* deletion/duplication was correlated with poor prognosis in Liver hepatocellular carcinoma (LICH) patients (LIHC-1867.3.3.N.3.0.126.1.4.12). *TP53* plays a crucial role in DNA repair and apoptosis (Blagih et al., 2020).

The link between *TP53* CNV changes and adverse outcomes in LIHC suggests its potential as a marker for high-risk patients. Loss of *TP53* function can weaken DNA repair and apoptosis, facilitating tumor progression. This underscores *TP53*’s role in restraining tumor aggressiveness, highlighting it as a potential therapeutic target. Exploring similar negatively correlated genes can reveal critical mechanisms in cancer suppression and inform targeted therapies.

Identifying genes that are negatively correlated with aggressive tumor features can highlight potential tumor suppressors or biomarkers of less aggressive disease. For example, reduced levels of E-cadherin (*CDH1*) are associated with increased invasiveness and metastasis in several types of cancer (Berx and Van Roy, 2001). We identified 16 signatures with *CDH1*, which is overexpressed in ten cancer types (PAAD, COAD, BRCA, LGG, HNSC, THYM, STAD, READ, GBM, and PRAD). In head and neck squamous cell carcinoma (HNSC) patients, *CDH1* mRNA expression was found to be negatively correlated with MSI (HNSC-814.6.2.N.3.0.0.2.2.3) (Supplementary Dataset S3). Notably, *CDH1* mutations in HNSC patients are rare (Supplementary Dataset S1T) compared to *TP53* mutations (Supplementary Dataset S1U and Supplementary Figure S9).

This negative correlation suggests that higher *CDH1* expression may contribute to tumor stability and cohesion, consistent with its role as a tumor suppressor and adhesion molecule. In HNSC, where high MSI frequently correlates with aggressive behavior and poor prognosis, elevated *CDH1* expression may help preserve cellular integrity, potentially limiting the tumor’s invasive capacity. This aligns with *CDH1*’s function in stabilizing cell-cell interactions and opposing epithelial-mesenchymal transition, a process often heightened in MSI-high tumors (Berx and Van Roy, 2001; Liu et al., 2017).

The presence of solo *CDH1* signatures across different cancer types further underscores *CDH1*’s potential as a marker of epithelial integrity and reduced invasiveness, especially in tumors with a preserved epithelial phenotype. Using *CDH1* as an indicator of cellular cohesion could improve patient stratification, identifying patients who may benefit from therapies focused on maintaining cell adhesion and curbing invasion-related pathways. This finding supports the need for further research into *the role of CDH1 in tumor stability and its potential as a biomarker across variou*s cancer types.

Thus, in contrast to models restricted to canonical forms of cell death, our 25-form RCD framework preserves the mechanistic and phenotypic diversity of RCD programs, offering greater resolution for identifying tumor-specific vulnerabilities and informing precision oncology strategies.

### 4.3 Prognostic and diagnostic potential of transcript-specific signatures

The transcript-level correlations observed for *MAPK10* underscore the importance of distinguishing specific isoforms in cancer research and clinical applications. While *MAPK10* as a gene locus shows variability across cancer types, individual transcripts reveal distinct associations with phenotypic features and patient outcomes. This specificity highlights the potential for isoform-level resolution to refine prognostic tools and therapeutic strategies. For instance, identifying protective or risk-associated transcripts can enhance the accuracy of patient stratification, allowing for more personalized treatment plans.

The distinct roles of *MAPK10* isoforms in tumor progression and interactions with the microenvironment also emphasize the need for targeted therapeutic approaches. By isolating isoforms associated with pro-tumoral or anti-tumoral phenotypes, therapies could be designed to selectively modulate these transcripts, maximizing treatment efficacy while minimizing off-target effects. This approach could be precious in cancers where *MAPK10* isoforms contribute differentially to immune infiltrates, such as “cold” or “hot” tumors, potentially guiding the selection of immunotherapy strategies.

The heterogeneity within *MAPK10* reinforces the importance of transcriptomics in understanding cancer biology. Whole-gene analyses may overlook critical isoform-specific contributions that drive tumor behavior and therapeutic response. As such, incorporating transcript-level data into clinical workflows could enhance diagnostic precision, prognosis accuracy, and the development of isoform-targeted therapies, representing a significant advancement in precision oncology.

### 4.4 Application and translational potential in clinical settings

Cancer therapies, including immunotherapy, aim to eliminate cancer cells, with their success often influenced by genes that regulate cell death. Most RCD-associated genes play either a pro-RCD or an anti-RCD role. However, depending on the cancer context, specific RCD-associated genes can promote or inhibit cell death, affecting their suitability as therapeutic targets. It is also known that some genes exhibit dual roles, acting as pro-RCD or anti-RCD agents based on the cancer type. For example, *SLC7A11*, which codes for a component of a sodium-independent, anionic amino acid transport system specific for cysteine and glutamate, promotes resistance to ferroptosis in gliomas (e.g., LGG-2956.3.3.N.3.0.0.2.4.4) but inhibits ferroptosis in endometrial carcinoma (e.g., UCEC-1106.2.1.P.3.2.0.2.4.4) (Fang et al., 2023; Zhu and Xu, 2024; Liu et al., 2020) (Supplementary Dataset S1V).

In cancer treatment, genes that promote RCD are often considered desirable targets because they facilitate the elimination of cancer cells. Conversely, genes that inhibit RCD can contribute to therapy resistance, making them challenging targets in specific cancers. Customizing therapeutic strategies based on the gene’s role in RCD within the specific cancer type can optimize treatment outcomes. Therapies should be aligned with whether a gene’s function is to promote or inhibit cell death, ensuring that the approach enhances the effectiveness of the treatment.

The RCD signature database holds significant promise for practical application in preclinical settings, offering valuable tools for patient stratification, personalized treatment plans, prognostic applications, and therapeutic decision-making. These signatures may enable categorizing patients based on their molecular profiles, leading to more tailored and effective treatment strategies.

**FIGURE 9:**
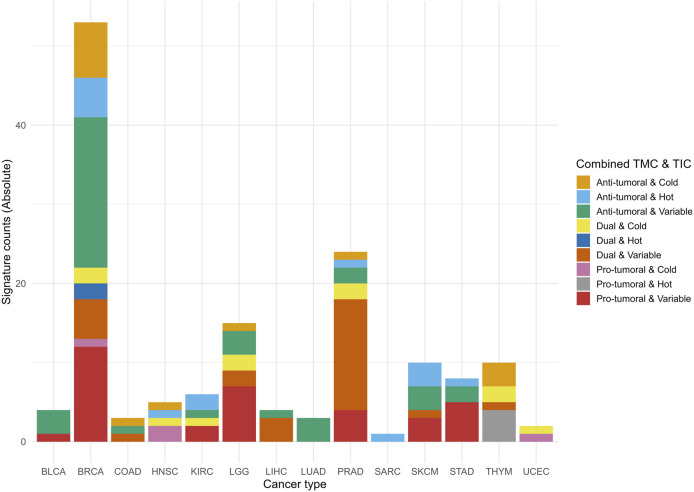
Accumulated histogram illustrating the distribution of mutation-specific signatures by meaningful immunotherapy potential across cancer types. The histogram shows the absolute counts of signatures associated with the combined Tumor Microenvironment Contexture (TMC) and Tumor-infiltrating lymphocyte contexture (TIC) ranks. Each bar represents a specific cancer type abbreviation (CTAB), and segments within the bars show the distribution of combined ranks categorized as Anti-tumoral & Hot, Dual & Variable, and Pro-tumoral & Cold, among others. The colors correspond to the Combined TMC and TIC ranks, mapped using the Okabe-Ito color palette extended for color-blind friendliness. Data were processed and summarized from multi-omic analyses of mutation-associated signatures with the potential for immunotherapy (Supplementary Dataset S1W).

We identified 148 gene signatures with somatic mutations positively correlated with TMB and with immunotherapeutic potential by their association with immune infiltrate profiles in fourteen cancer types (Supplementary Dataset S1W). The distribution of these mutation-specific signatures by cancer type and their immunotherapeutic potential is shown in Figure 9. Mutations in these genes are likely sources of neoantigens, as high TMB produces more immunogenic mutations (Zhang et al., 2024). This connection suggests the mutation-specific signatures could identify neoantigen targets for personalized therapies, such as cancer vaccines or T cell-based treatments.

The practical application of our findings lies in stratifying patients by using the signatures as prognostic tools to guide therapeutic decisions based on the cancer’s molecular profile (Wang D. R. et al., 2022). To bring our multi-omic signature database into clinical practice, it is essential to conduct rigorous clinical trials that validate both its efficacy and reliability. This involves evaluating the predictive power of the signatures across diverse patient cohorts and confirming reproducibility in different clinical settings (Wang D. R. et al., 2022). Notably, cross-referencing the gene components in the database with existing literature reveals that some signatures or their members have already been evaluated in previous preclinical studies, which highlights the translational potential of our findings, bridging preclinical insights with clinical applications. Out of the 150 widely recognized immunological targets in cancer research, 91 (60,7%) are included in the signatures identified in this study (Supplementary Dataset S1L).

### 4.5 Cases of clinically validated RCD multi-omic signatures

We identified 879 multi-omic signatures (Supplementary Dataset S10) that contain at least one gene member from 27 out of 29 genes whose protein products are classified as chimeric antigen receptor (CAR) targets and are currently under investigation in clinical trials as identified by Clinicaltrials.gov (Dannenfelser et al., 2020).

We exemplify the translational impact of the RCD multi-omic signature database with two cases in which members of the multi-optosis signatures have been clinically validated in independent studies. The first case is *CD274* (a driver gene that encodes for PD-L1) (Topalian et al., 2012). The finding that *CD274* is involved in eight RCD processes (apoptosis, autophagy, cuproptosis, efferocytosis, ferroptosis, necroptosis, pyroptosis, and necrosis) expands our understanding of the multifaceted roles of PD-L1 in cancer biology (Supplementary Dataset S1B). This broad involvement suggests that PD-L1 may influence tumor progression and response to therapy through multiple pathways, not just immune evasion. This knowledge can lead to targeted and effective therapeutic strategies that address these various pathways.

The positive correlation between *CD274* mutations and TMB in GBM-410.2.1.P.3.35.0.4.4.8, LGG-1442.2.1.P.3.35.0.3.4.8, and PAAD-773.2.1.P.3.42.0.2.4.8 suggests that higher TMB, often associated with better responses to immunotherapy, is linked to the occurrence of *CD274* mutations (Supplementary Figure S10, Supplementary Dataset S1X). Given the low frequency of *CD274* somatic mutations in those cancer types (0.4%; 4 mutations in 998 patients, Supplementary Dataset S1X), as compared to *TP53*, a prominent driver cancer gene (Supplementary Dataset S1Y), the findings highlight that even rare mutations can have significant clinical implications. Patients with high TMB are more likely to have neoantigens that enhance the immune response, making them better candidates for immunotherapy. This correlation can guide the selection of patients for immune checkpoint inhibitor therapies, potentially leading to better clinical responses.

The identification of *CD274* mutations as a risk factor in at least one survival metric in patients with GBM, LGG, and PAAD (Supplementary Figure S9) aligns with previously reported associations (Zeng et al., 2023; Chen et al., 2018; Fen et al., 2017). It reinforces the role of PD-L1 as a critical biomarker for patient stratification in these cancers. Clinicians can assess PD-L1 expression levels to identify patients more likely to benefit from PD-L1/PD-1 checkpoint blockade therapies, thus personalizing treatment plans and improving outcomes.

Understanding the diverse roles of *CD274* in various RCD processes (apoptosis, autophagy, cuproptosis, efferocytosis, ferroptosis, necroptosis, pyroptosis, and necrosis) (Supplementary Dataset S1B) and its positive association with TMB can inform the development of combination therapies. For instance, combining PD-L1 inhibitors with agents targeting specific RCD pathways (i.e., ferroptosis inducers or necroptosis inhibitors) could enhance therapeutic efficacy by simultaneously disrupting multiple tumor survival mechanisms.

The second case example is the *AXL* receptor tyrosine kinase, which plays critical roles in cellular functions such as growth, migration, aggregation, and anti-inflammation in multiple cell types (Goyette and Cote, 2022), and it is term-based associated with apoptosis, efferocytosis, necroptosis, and necrosis in various cancer types (Supplementary Dataset S1B). Our findings show that *AXL* mRNA expression is negatively correlated with stemness in PAAD-718.6.3.N.3.44.43.3.4.4, LGG-1367.6.3.N.3.35.35.2.4.4, and STAD-1167.6.3.N.3.35.1.3.2.4 patients (Supplementary Dataset S3). Specifically, these signatures indicate that high *AXL* expression is linked to decreased stemness in these cancers. Conversely, in PAAD and STAD patients, *AXL* mutations show a positive correlation with TMB (Supplementary Dataset S3). The frequency of *AXL* somatic mutations in those cancer types is low (5.5%; 34 mutations in 616 patients, Supplementary Dataset S1Z) compared to *TP53* (Supplementary Figure S9, Supplementary Dataset S1AA). Thus, adverting that even infrequent mutations can have significant clinical implications. Notably, the overexpression of *AXL* in these three cancer types is a risk factor across three to four metrics of patient survival (Supplementary Dataset S3).

Cross-referencing shows that anti-human monoclonal antibodies targeting the AXL receptor tyrosine kinase inhibit AXL activity effectively, limiting the proliferation and migration of pancreatic cancer cells *in vitro* and *in vivo* (Leconet et al., 2014). This evidence suggests a promising approach for immunotherapy in PAAD, LGG, and STAD patients, underscoring the potential for these signatures to inform innovative therapeutic strategies involving anti-AXL antibodies and small molecule AXL kinase inhibitors. Dysregulated *AXL* expression in STAD is highlighted as a promising therapeutic target, further supporting the relevance and potential impact of targeting AXL in gastrointestinal cancers (Pidkovka et al., 2023).

### 4.6 Validation in PRECOG cancer types

While 73 of the 126 mRNA-specific signatures were successfully validated in PRECOG cancer types equivalent to TCGA (Lung adenocarcinoma, Breast cancer, Glioma, Astrocytoma, and Prostate cancer), the validation rate (58.4%) highlights important biological and technical considerations. Several factors may explain why not all signatures showed consistent prognostic value across the independent PRECOG dataset: (1) lack of equivalent TCGA *versus* PRECOG cancer type (example: CESC - Cervical squamous cell carcinoma and endocervical adenocarcinoma; n = 5 signatures); (2) gene absent in PRECOG (example: ADAMTS9-AS1 in PRAD-1064.6.3.N.2.95.26.1.2.1); (3) the validation process relied on stringent statistical thresholds (|Meta-Z| > 3.09 or < −3.09, p < 0.001) to identify significant poorer or better prognosis, respectively. Signatures with weaker but still biologically relevant effects may not have met these thresholds in PRECOG, leading to their exclusion from the validated set. (4) Some signatures may exhibit cancer-specific prognostic value, meaning they are highly relevant in certain cancer types but not others. While PRECOG includes cancer types equivalent to TCGA, the absence of certain subtypes or including additional subtypes in PRECOG could explain why some signatures were not validated. (5) For multi-gene signatures, the median meta-Z score was computed across all genes, which may dilute the contribution of individual genes with strong prognostic effects. This aggregation approach could cause the loss of signal for signatures where only a subset of genes drives the prognostic association.

Despite these challenges, the validation of 73 signatures in PRECOG underscores their robustness and clinical relevance across independent datasets. The validation rate highlights the complexity of translating gene expression signatures into universally applicable prognostic tools and emphasizes the need for further refinement and context-specific validation in future studies.

### 4.7 Advanced tools for RCD data analysis

Although existing resources provide valuable insights, they have limitations that our model addresses. Four comprehensive and interactive online tools are currently available to support research on RCD in cancer: RCD map^10^ (Ravel et al., 2020), FerrDb^11^ (Zhou and Bao, 2020), HAMdb^12^ (Wang et al., 2018), XDeathDB^13^ (Gadepalli et al., 2021) and RCDdb^14^ (Wang et al., 2024). The first appears to have inactive hyperlinks. FerrDb is dedicated to ferroptosis regulators and disease associations. It categorizes regulators into genes (drivers, suppressors, markers, unclassified) and substances (pure and mixtures like iron, erastin, and herbal extracts). These are further classified as inducers or inhibitors. FerrDb includes seven curated datasets. HAMdb is a database of autophagy modulators and their disease links, containing 796 proteins, 841 chemicals, and 132 miRNAs. It helps identify new modulators, drug candidates, and therapeutic targets through a user-friendly interface for easy searching and browsing, advancing autophagy research in cancer and other diseases. XDeathDB gathers information about a 12-optosis model that includes intrinsic apoptosis, autosis, efferocytosis, ferroptosis, immunogenic cell death, lysosomal cell death, mitotic cell death, mitochondrial permeability transition, necroptosis, parthanatos, and pyroptosis. It integrates big data for cell death gene-disease associations, gene-cell death pathway associations, pathway-cell death mode associations, and cell death-cell death associations derived from literature reviews and public databases. RCDdb features over 3,000 literature-derived annotations covering 1,850 RCD-associated genes linked to 15 RCD forms (apoptosis, pyroptosis, necroptosis, autophagy-dependent cell death, entotic cell death, NETotic cell death, parthanatos, MPT-driven necrosis, immunogenic cell death, lysosome-dependent cell death, ferroptosis, alkaliptosis, oxeiptosis, cuproptosis, and disulfidptosis). It integrates data on diseases, drugs, pathways, proteins, and gene expression and provides advanced visualization tools and three analytical modules to enable users to identify and study RCD-related features.

The RCDdb is the first comprehensive, manually curated database focused on annotating and analyzing the 15 known RCD forms.

Despite their comprehensive scopes, FerrDb, HAMdb, XDeathDB and RCDdb do not index outputs by significance, making it challenging to prioritize critical associations, which can hinder effective data utilization and research prioritization. We developed the *CancerRCDShiny* web browser and the *Cancer Regulated Cell Death Data Analyst* tools to address these gaps. These new tools enhance the utility and impact of our findings, making them more accessible and actionable for researchers and clinicians. Their integrative and user-friendly design facilitates efficient extraction, analysis, and visualization of RCD data in cancer, ultimately advancing our understanding and treatment of cancer through more precise biomarkers and targeted therapies.

## 5 Shortcomings and limitations

This study has shortcomings and limitations that should be considered when selecting impactful signatures. First, the gene inventory is an ongoing effort, which means some genes reported in various studies may have been omitted since our catalog is primarily based on the NCBI Gene database. Despite our cross-referencing, relying on a single database means the catalog may not comprehensively include all genes associated with RCD forms reported in the literature.

Second, using a stringent genome-wide significance threshold (*padj*-value < 5 × 10^-8) while minimizing false positives may reduce sensitivity, especially in smaller datasets or those with lower signal-to-noise ratios. Users should consider the specific context of their dataset and study design when applying this threshold. A less stringent threshold might enhance sensitivity in particular scenarios while maintaining the stringent threshold is crucial in larger datasets to control false discovery rates. We have made our source code publicly available, enabling researchers to fine-tune the significance threshold.

Third, the signatures identified in this study are related to primary tumor samples. Therefore, the impact values of these signatures in recurrent tumors, metastatic tumors, and primary blood-derived cancers were not addressed in this study. Future studies should expand the analysis to these other tumor types to provide a more comprehensive understanding of the signatures’ roles across different cancer stages and contexts.

While our model currently includes seven multi-omic layers with broad Pan-Cancer coverage from TCGA, epitranscriptomic modifications such as N^6^-methyladenosine (m^6^A) were not included due to the absence of high-resolution, uniformly processed m^6^A data across cancer types. As such datasets become more widely available, future iterations of the CancerRCDShiny framework will seek to incorporate m^6^A and related RNA modifications to further refine isoform-level phenotypic associations.

Fourth, the corpora of PDFs comprise only free-access full-text files. This limitation may cause a biased dataset, as some relevant studies published in subscription-based journals were not included. Cross-referencing gene targets and gene components of signatures might miss critical information available in those restricted-access publications. Future research should incorporate a broader range of manually curated sources to ensure greater accuracy and depth in the findings.

Lastly, although this study identifies biomarkers with potential immunotherapeutic applications, it does not incorporate AI-driven drug discovery or molecular docking methods to identify or validate therapeutic compounds. Such approaches could refine our ability to screen for specific inhibitors or activators targeting RCD-related pathways and enhance the translational relevance of our findings. Future research should aim to integrate AI and docking-based platforms into the *CancerRCDShiny* tool to support the discovery of novel drugs targeting the multi-omic signatures identified in this study.

## 6 Strengths

This study offers a comprehensive analysis of 25 forms of RCD in cancer, integrating seven multi-omic layers to identify biologically grounded and clinically relevant signatures. A structured scoring system was implemented to assess signature significance, supported by a PDF-AI-based literature mining strategy for evidence-based validation. The Multi-Optosis framework was intentionally designed to preserve the biological and phenotypic heterogeneity inherent to multi-omic cancer data, rather than reducing the complexity of over 44,000 multi-layered signatures into meta-signatures. This approach enables context-specific interrogation across omic layers, phenotypic attributes, and tumor types. By systematically integrating RCD forms with phenotypic and survival traits across multiple cancers, the model establishes a structured and reproducible platform for biomarker discovery and therapeutic target prioritization. The adoption of a distinct signature nomenclature and the implementation of an interactive Shiny application (*CancerRCDShiny*) further distinguish this resource from general-purpose Pan-Cancer studies by providing biologically coherent and clinically interpretable outputs.

## 7 Concluding remarks

This study introduces the multi-optosis framework as a novel, integrative approach for investigating RCD mechanisms in cancer. By incorporating 25 distinct forms of RCD, the model transcends traditional, single-pathway analyses, offering a holistic view of the intricate crosstalk between RCD pathways. This framework advances our understanding of cancer progression and treatment resistance while providing a robust platform for identifying genome-wide biomarkers and actionable therapeutic targets. Notably, the multi-optosis model lays the foundation for clinical applications, such as stratifying patients based on RCD-related phenotypes and designing therapies that target multiple RCD pathways for enhanced efficacy.

We developed a signature database enriched with a systematic nomenclature that reveals hidden correlations between multi-omic and phenotypic features. Our ranking method ensures a balanced and comprehensive assessment of signature relevance by integrating survival metrics and tumor immune infiltration profiles. This prioritization speeds up the discovery of actionable insights and supports the development of personalized therapeutic strategies to improve patient outcomes.

Practical applications of our findings are facilitated by user-friendly tools such as CancerRCDShiny and the Cancer Regulated Cell Death Data Analyst. These tools enable researchers and clinicians to explore RCD multi-omic signatures efficiently, leveraging dynamic visualization and customizable reporting capabilities to enhance data interpretation.

By addressing the complexity and heterogeneity of cancer biology, the multi-optosis framework provides a detailed understanding of RCD gene associations in cancer progression and resistance. This integrative approach paves the way for identifying candidate biomarkers and therapeutic targets, driving the development of more effective cancer treatments.

Together, the multi-optosis model and its associated tools represent a significant advancement in cancer biomarker discovery and translational research, offering invaluable resources for personalized cancer therapies and improved clinical outcomes.

## Data Availability

All source codes for these analyses are in the supporting source code repository available at GitHub URL: https://github.com/CancerRCD.

## Footnotes

1 UCSCXena: https://xena.ucsc.edu/

2 UCSCXenaShiny: https://shiny.zhoulab.ac.cn/UCSCXenaShiny/#

3 PDF AiDrive: https://myaidrive.com

4 GTEx Portal: https://www.gtexportal.org/

5 BioMart: https://www.ensembl.org/biomart/

6 R software package: http://www.R-project.org

7 DGIdb: https://dgidb.org

8 PRECOG: https://precog.stanford.edu/

9 COSMIC: https://cancer.sanger.ac.uk/cosmic

10 RCD map: https://navicell.vincent-noel.fr/pages/maps_rcd.html

11 FerrDb: http://www.zhounan.org/ferrdb/legacy/index.html

12 HAMdb: http://hamdb.scbdd.com/

13 XDeathDB: https://pcm2019.shinyapps.io/XDeathDB/

14 RCDdb: http://chenyclab.com/RCDdb/
